# Biomolecule Conjugation Strategy for HAGM Cryogels to Create 3D Immune Niches that Induce Multifunctional T Cells

**DOI:** 10.1021/acsbiomaterials.5c00134

**Published:** 2025-07-19

**Authors:** Marjolein Schluck, Jorieke Weiden, Roel Hammink, Lea Weiss, M. Eloisa Vega Quiroz, Maren Pfirrmann, Laia Junquera Guinovart, Vincent van der Steen, Chadia Archidi, Leanne H. Minall, René Classens, Mahboobeh Rezaeeyazdi, Thibault Colombani, Sidi A. Bencherif, Carl G. Figdor, Martijn Verdoes

**Affiliations:** † Department of Medical BioSciences, Radboud University Medical Center, Nijmegen 6525 GA, The Netherlands; ‡ Oncode Institute, Utrecht 3521 AL, The Netherlands; § Institute for Chemical Immunology, Utrecht 3584 CH, The Netherlands; ∥ Department of Chemical Engineering, 1848 Northeastern University, Boston, Massachusetts 02115, United States; ⊥ University Rouen Normandie, CNRS, PBS UMR 6270, Rouen F-76000, France

**Keywords:** T cells, scaffolds, bioconjugation, 3D immune niche, cancer immunotherapy

## Abstract

Recently, biomaterials have emerged as tools to activate and expand T cells in the context of cancer immunotherapy. Most designs accommodate T cells with a stimulatory two-dimensional (2D) environment. In contrast, three-dimensional (3D) scaffolds, mimicking the complex architecture of the lymph node, have been shown to outperform 2D synthetic constructs, resulting in a more optimal T-cell expansion and phenotype. Here, we used injectable glycidyl methacrylated hyaluronic acid (HAGM)-based cryogel scaffolds to create a modular biodegradable 3D stimulatory immune niche. We developed a strategy to achieve highly specific and efficient covalent linking of immune-activating biomolecules, such as T-cell-activating peptide MHC complexes and antibodies, to HAGM scaffolds without compromising the injectable properties of the cryogels. Importantly, because our conjugation strategy is carried out postcryogelation, biomolecules are not exposed to free radicals and freeze–thawing cycles, facilitating highly reproducible covalent attachment. Our scaffold potently activates human- and murine-T cells, inducing higher levels of multifunctional T cells with a less exhausted phenotype compared to 2D cultures. Following injections, HAGM scaffolds retain up to 60% of highly proliferative T cells. In conclusion, our HAGM scaffolds are an easily adaptable tool for robust T-cell activation, thus further expanding the biomaterial-based immunotherapy toolbox.

## Graphical abstract

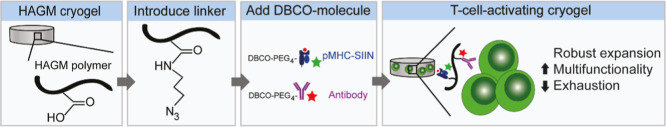

## Introduction

Immunotherapy has dramatically changed cancer treatment by aiming to eradicate tumor cells by strengthening an anticancer immune response.^1^ T-cell-based immunotherapies focus on expanding and activating tumor-reactive T cells to evoke T-cell-mediated tumor cell killing. To this end, cancer patients are infused with high numbers of potent T cells via adoptive T cell therapies (ACT).^2–5^ T-cell-based immunotherapies have demonstrated remarkable therapeutic efficacies in the treatment of various cancers, including melanoma^6–11^ and B cell malignancies.^2,12–15^ Despite these promising results, there are still hurdles to overcome, which include the laborious manufacturing processes, variability in treatment efficacy across patients, poor T-cell persistence in vivo following infusion, and off-target effects resulting in neurological toxicity and cytokine release syndrome.^16–21^

Recently, biomaterial-based strategies have shown promising results in overcoming these hurdles.^22,23^ Biomaterials in the form of artificial antigen-presenting cells (aAPCs) have been developed to bypass the use of autologous monocyte-derived dendritic cells (moDCs) for T cell stimulation and expansion. aAPCs are based on synthetic constructs that mimic natural APCs by presenting T-cell-activating signals, such as agonistic antibodies against CD3 (αCD3) or peptide major histocompatibility complexes (pMHC)^24,25^ to provide T-cell receptor (TCR) stimulation (signal 1) to the T cells. To achieve proper T-cell functionality, agonistic antibodies against costimulatory molecules (signal 2) such as CD28, 4-1BB, or OX-40 are provided.^26–30^ Modification of the biomaterial with cytokines (signal 3)^31,32^ or supplementation of soluble cytokines to the culture provides the T cells with additional survival signals. In addition to aAPCs for the ex vivo expansion of T cells, several biomaterial-based designs have focused on either enhancing in vivo persistence of T cells^33,34^ or on the in vivo stimulation and activation of T cells,^24,35,36^ omitting the need for laborious ex vivo T-cell expansion.

Most of these biomaterial designs accommodate T cells with a stimulatory two-dimensional (2D) environment, a context that is not representative of the complex three-dimensional (3D) structure of the lymph node where natural APCs and T cells normally interact. Providing T cells with a 3D stimulatory environment indeed was found to improve T-cell activation and expansion.^37,38^ Additionally, 3D scaffolds have been employed as supportive environments for the ACT of T cells in vivo. These scaffolds provide sustained availability of stimulatory cues such as agonistic antibodies and stimulatory cytokines such as IL-2 or IL-15 to expand T cells, thereby creating supportive synthetic immune niches.^39–41^ Furthermore, scaffolds can also act as in vivo depots to deliver and disperse antigen-specific T cells.^42^ Despite their functionality, many existing 3D scaffolds are nonbiodegradable^41,43–46^ or noninjectable,^40,41,46–48^ which requires surgical procedures for implantation in the body. Alternatively, many available injectable materials often rely on shear thinning or in situ gelling of the material during or after injection, but these approaches do not allow for structurally and mechanically defined scaffolds after introduction into the body.^39,49,50^ Finally, the biodegradability of the materials used for the scaffold is of importance for their application. This emphasizes the need for improved scaffold designs.

Here, we exploit hyaluronic acid (HA), a naturally occurring component of the extracellular matrix, to prepare biocompatible and biodegradable 3D scaffolds.^51^ To this end, HA is modified with glycidyl methacrylate (GM) to form glycidyl methacrylated HA (HAGM) which can cryopolymerize into 3D scaffolds. The HAGM scaffolds are macroporous, possess a high degree of pore connectivity, are mechanically robust, and exhibit unique deformability, which enables minimally invasive delivery of these preformed scaffolds by injection through a hypodermic needle.^52–54^ In this study, we developed a versatile and straightforward strategy to covalently couple biomolecules to HAGM cryogels postcryogelation, preventing exposure of the biomolecules to the free radicals present during cryopolymerization and freeze–thawing, which could potentially denature proteins. To create synthetic 3D T-cell-stimulatory immune niches, we conjugated stimulatory monoclonal antibodies (mAbs) and pMHC to the HAGM cryogels. Our results highlight that the presence of comonomers during cryogelation is required for the labeling of preformed HAGM cryogels. Moreover, we show that the synthetic 3D T-cell-stimulatory immune niches can potently activate human and murine T cells. Additionally, comparison of T-cell activation in the 3D immune niche to that in 2D cultures indicated higher levels of multifunctional T cells with a less exhausted phenotype in the 3D culture environment. Taken together, our novel conjugation strategy for the attachment of biomolecules to HAGM scaffolds provides a highly versatile platform for efficient T-cell stimulation in a 3D microenvironment.

## Materials and Methods

### Reagents and Antibodies

For the preparation of the stimulatory biomolecules, the following mAbs were used: antihuman CD3 (clone OKT3), antihuman CD28 (clone 9.3), and antimouse CD28 (clone 37.51), all from BioXcell. The amounts of T-cell stimulating biomolecules used are indicated in the Figures or Figure legends. The T cell phenotyping panels and gating strategies are depicted in Figure S5. For flow cytometry analysis, αCD4 PerCP (300528), αCD8 BV510 (344732), αCD95 BV421 (305624), αCD45RA BV510 (304142), αPerforin A647 (308110), αGranzyme B PE (372208), αIL-2 PerCP-Cy5.5 (500322), αCD39 BV421 (328214), αTIM-3 APC (345012), αCD8 PerCP (344708), αCD4 PerCP (300528), and αCD25 PE-Cy7 (302612) were purchased from BioLegend. αCD4 APC-Cy7 (557871), αIFNγ BV421 (562988), αTIGIT BB700 (747846), αPD-1 BV510 (563076), and αCLTA-4 PE (555853) were obtained from BD Biosciences. αCD4 PE-Cy7 (25-0047-42) and αTNF-α PE-Cy7 (25-7349-82) were obtained from eBioscience, and αCCR7 A647 (130-099-363) was obtained from Miltenyi.

### Preparation of Hyaluronic Acid–Based Cryogels

Methacrylated hyaluronic acid (HAGM) was prepared by reacting hyaluronic acid (HA), either high molecular weight (HMW) (1.6 × 10^6^ Da) or LMW (0.36 × 10^6^ Da) hyaluronic acid sodium salt from streptococcus equi (Sigma-Aldrich), with glycidyl methacrylate (Sigma-Aldrich) as previously reported.^51^ Briefly, 3 g of HA was dissolved in 600 mL of phosphate-buffered saline (PBS, pH 7.4), and 200 mL of dimethylformamide (DMF) was added. Next, 27.5 μL (20 mg) of triethylamine (TEA, Sigma-Aldrich) was mixed in slowly, after which 50 g of GM was added. After 10 days of stirring at room temperature (RT), the solution was precipitated in a large excess of acetone, filtered, and dried in a vacuum oven overnight. The degree of methacrylation of HAGM (1 mg/mL in deuterium oxide (D_2_0, Merck)) was determined using high-resolution ^1^H NMR spectra (Bruker Avance III 400 MHz, Figure S2a). The efficiency of methacrylation was determined based on the ratio of the integrals for HA protons to the methylene protons of GM using MestReNova software.

To produce macroporous HAGM cryogels, HAGM was dissolved in Milli-Q (MQ) water [3% (wt/vol)], and free-radical polymerization of this prepolymer solution was induced by the addition of tetramethyl ethylenediamine (TEMED, Sigma-Aldrich, [0.15% (wt/vol)]) and ammonium persulfate (APS, Sigma-Aldrich, [0.25% (wt/vol)]). For all experiments, HMW HAGM was used unless indicated otherwise in the figure legend. As indicated, GM or hydroxypropyl methacrylate (HPMA, Sigma-Aldrich) was added before polymerization as comonomers. Cross-linking was performed for 17 h at −20 °C in a Teflon-mold with 4 × 4 × 1 mm^3^ dimensions precooled at 4 °C to obtain 16 μL cryogels. Cryogels were thawed for 15 min at RT prior to labeling. As the addition of the comonomer HPMA increased the labeling of the HAGM cryogels and their stability, all batches used throughout this study were supplemented with HPMA with concentrations ranging from 0.8% (wt/vol) to 2.2% (wt/vol) to account for differences between HAGM batches used.

### Characterization of HAGM Cryogels

Fluorescence microscopy was performed to evaluate the HAGM polymer network, pore size, and the distribution of fluorophore-functionalized antibodies (Olympus FV1000 Confocal Laser Scanning Microscope and Zeiss LSM900), and images were analyzed with ImageJ 1.51s (Fiji) software.^55^ Average pore size of the cryogels was obtained by manually measuring the pore diameter of rhodamine-labeled poly- *l* -lysine (Nanocs) stained cryogels ([0.1% (wt/vol)] for 1 h at RT) for at least 30 pores per cryogels. Injectability of 4 × 4 × 1 mm HAGM cryogels was assessed via injection through 16G needles together with 200 μL PBS. The swelling ratio was determined on 8 × 5 mm^2^ and pore connectivity on 16 × 1 mm^2^ cylindrical HAGM cryogels as previously reported.^52^

### Production of pMHC Complexes

Refolding of peptide major histocompatibility complexes (pMHC) was based on the reported methods^56,57^ to obtain mouse H-2K^b^/SIINFEKL complexes. Mouse H-2K^b^ and human β2 microglobulin (β2m) were produced in (sequences in Figure S6). Inclusion bodies were isolated, purified, and solubilized in denaturation buffer (8 M urea in 100 mM Tris–HCl (pH 8)). β2m was prefolded in dialysis against PBS buffer. To refold MHC class I complexes, prefolded β2m (2 μM final concentration) and solubilized H-2K^b^ (1 μM final concentration) were added dropwise to short peptide antigen OVA_257–264_ (SIINFEKL, GenScript) dissolved in refolding buffer at 60 μM. Refolding reactions were incubated at 10 °C for 4–5 days. Reaction mixtures were then spun down, filtered (to remove aggregates), and refolding reactions were concentrated using Amicon centrifugal filters (30 kDa cutoff, Merck). pMHC complexes were purified via size exclusion on a Cytiva HiLoad 16/600 column in a Bio-Rad NGC medium pressure chromatography system. Fractions were evaluated for the presence of pMHC complexes using SDS-PAGE gel electrophoreses, and fractions of interest were pooled and concentrated on Amicon centrifugal filters (30 kDa cutoff, Merck). Protein concentration was calculated using absorbance at 280 nm on a NanoDrop 2000c spectrophotometer (ThermoFisher Scientific) with respect to the molecular extinction of H-2K^b^/SIINFEKL (94770 M-1 cm-1). pMHC complexes were snap-frozen in liquid nitrogen and stored at −80 °C.

### Functionalization of Biomolecules with DBCO and Dye

αhCD3, αhCD28, αmCD28, and pMHC-SIINFEKL were functionalized with DBCO-PEG4-NHS (click chemistry tools) and Atto488-NHS (hαCD3, atto-tec), Alexa Fluor 647-NHS (αhCD28 and αmCD28, ThermoFisher Scientific), and Alexa Fluor 594-NHS (αhCD28, ThermoFisher Scientific) as described before.^32^ Typically, this reaction yielded biomolecules with an average degree of labeling of 1–3 DBCO and 1–3 dyes per biomolecule.

### Preparation of Azido-HAGM Cryogels

All reaction equivalents that are presented are relative to the estimated number of carboxylic acids present in the HAGM cryogels. HAGM cryogels were labeled with DBCO-functionalized dye-labeled biomolecules. First, carboxylic acids on HAGM polymers were activated by incubation with 10 equiv of EDC and 10 equiv of NHS in 50 μL of MES buffer (100 mM, pH 5.5) for 1 h at RT on a shaker (160 rpm). Cryogels were washed twice with 200 μL of MES buffer (100 mM, pH 5.5) and twice with 200 μL of MQ. Next the cryogels were incubated for 2–5 h in borate buffer (pH 8.9, 120 mM) with 0.5 equiv of azido-propylamine (Jena Biosciences). Finally, cryogels were washed consecutively with dimethyl sulfoxide (DMSO, CryoSure) and PBS pH 7.4 before drying the cryogels with a gauze to remove all excess liquid. To study acylation efficiency, the carboxylic acids of 4 × 4 × 1 mm^3^ HAGM cryogels were activated with EDC/NHS and reacted with a Cy5-labeled linker, which was prepared by reacting DBCO-sulfo-Cy5 (1.2 equiv, Jena Bioscience) with azido-propylamine (500 μM) overnight at 37 °C in 0.1 M sodium bicarbonate (pH 8.5). The Cy5-labeled linker was added at 7 × 10^–3^ equivalents compared to the available carboxylic acids of 4 × 4 × 1 mm^3^ HAGM cryogels and incubated on a shaker at 37 °C for 1.5–2.5 h. Cryogels were washed twice with DMSO, followed by incubation with DMSO for 10 min at 37 °C and washed twice with MQ. Gels were resuspended in 100 μL of MQ and measured with the fluorescence plate reader (Tecan, Spark M10) to quantify fluorescence intensity.

### Labeling of Azido-HAGM Cryogels with DBCO-Conjugated Biomolecules

DBCO-conjugated biomolecules were added to the azido-HAGM cryogels in 100 μL of PBS at varying amounts and incubated overnight at RT while rolling. The next day, cryogels were washed five times with PBS and resuspended in 100 μL of PBS. The Tecan Spark M10 plate reader was used to measure the fluorescence intensity of labeled cryogels with the Magellan data processing and analysis software. This was compared with the fluorescence of a 12-point calibration curve for which the different biomolecules were added to HAGM cryogels in 100 μL of PBS without washing to calculate the respective protein concentrations in the cryogels. Moreover, the supernatants of the cryogels were measured and compared to a 12-point calibration curve of the biomolecules dissolved in PBS.

### T-Cell Purification

Total human CD3^+^ T-cell populations were isolated from buffy coats of healthy donors using Ficoll density gradient centrifugation (lymphoprep, Eliteck group). T cells were isolated from PBLs from healthy donors using the human PAN T-cell isolation kit according to manufacturer’s protocol (Miltenyi Biotec).

CD8α^+^ T cells were isolated from 6–12-week-old female or male OT-I mice (C57BL/6-Tg (TcraTcrb)­1100Mjb/Crl, Charles River) or WT mice (C57BL/6). Spleen and inguinal and axillary lymph nodes were digested for 30 min at 37 °C with DNase I (20 μg/mL, Roche) and Collagenase III (1 mg/mL, Worthington) and meshed over a 100 μm cell strainer. Splenocytes were treated with ammonium-chloride-potassium (ACK) lysis buffer for 3–5 min at RT to lyse blood cells. CD8α^+^ T cells were isolated using the mouse CD8α^+^ T-cell isolation kit (Miltenyi Biotec) according to protocol. All mice were housed at the Central Animal Laboratory (Nijmegen, The Netherlands) where food and water were provided ad libitum. This study was carried out in accordance with European legislation. Protocols were approved by the local authorities (CCD, The Hague, The Netherlands) for the care and use of animals with related codes of practice.

### Cell Culture

Isolated human T cells were cultured in X-vivo medium supplemented with 2% human serum for culture up to 3 days or in X-vivo medium supplemented with 4% human serum supplemented with IL-2 (30 U/ml) for culture up to 14 days. Isolated (OT-I) CD8α^+^ T cells were cultured in RPMI 1640 (Gibco) medium containing 10% fetal bovine serum (FBS), 0.5% antibiotic–antimycotic (Gibco), 0.1% 2-mercaptoethanol (50 mM in DPBS, Gibco), and 1% (2 mM) l-glutamine (Gibco) (full RPMI medium). When T cells were cultured for longer periods of time to study their phenotype for up to 14 days in culture, we refreshed the media every 3 to 4 days. Furthermore, cells in media and plate-bound conditions were split on days 5 and 9. Conditions with HAGM cryogels were transferred from a 96-well to a 48-well plate with 300 μL of media by day 5 and to a 24-well plate with 500 μL of media by day 9.

### T-Cell Stimulation Assays and Harvesting for Flow Cytometry

For proliferation assays, the total CD3^+^ T-cell population was stained with the proliferation dye Cell Trace Violet (2.5 μM, Invitrogen) in PBS containing 1% FBS for 10 min at 37 °C. Cryogels were washed three times with PBS, followed by three washes with media, and dried with a sterile 5 × 5 Surgical Care HG Compress (Klinion) under sterile conditions. The dehydrated gels were moved to a sterile flat-bottom 96-well plate (Corning). T cells were added in 16–20 μL to each cryogel. After 0.5–2 h incubation at 37 °C, an additional 180 μL of prewarmed medium was added to the cell-seeded gels, and they were incubated at 37 °C for the duration of the assay. For the proliferation experiment, 50 × 10^3^ cells were added per cryogel, and for the long-term assay, 100 × 10^3^ cells were added per cryogel. As a positive control, human total CD3^+^ T cells were cultured with plate bound αhCD3 (1 μg/mL)/αhCD28 (5 μg/mL). Supernatant was retrieved after 24 h of incubation for analysis of cytokine production using standard ELISA kits according to manufacturer’s protocol (Invitrogen). To determine intracellular cytokine production, human T cells were stimulated with PMA (20 ng/mL) and ionomycin (1 μg/mL) for 5.5 h in the presence of brefeldin A (Sigma-Aldrich, 10 μg/mL) and monensin (1:1000, 00-4505-51 eBioscience).

T cells were harvested from the HAGM cryogels by placing the cryogels in a flat-bottom 96-well plate and incubated with cold PBS + 2 mM ethylenediaminetetraacetic acid (EDTA, pH 8.0) for 10 min followed by centrifugation (200*g* for 10 min and 450*g* for 2 min at 4 °C). Cryogels were discarded, and cells were retrieved from the bottom of the well. The cells were stained with Fixable Viability dye eFluor 780 (BD Pharmingen), followed by cell surface staining. For the exhaustion panel and the effector panel, cells were permeabilized and fixed with the BD cyotfix/cytoperm kit following the manufacturer’s protocol and stained intracellularly. Flow cytometric analysis was performed on the BD FACS Verse, and data was analyzed using the FlowJo software, version 10.0.7.

To calculate the division index (DI) of the T cells, eq 1 was used

1 $$DI=\frac{\sum_{0}^{i}iX\frac{N_{i}}{2^{i}}}{\sum_{0}^{i}\frac{N_{i}}{2^{i}}}$$

in which *i* represents the proliferation peak, and *N* the percent of T cells in this specific proliferation peak.^58^

To determine the long-term expansion, 25 μL Precision counting beads (BioLegend) were added to the samples prior to FACS analysis. Cell numbers were determined using eq 2

2 $$absolute\;cell\;count\;\left(\frac{cells}{\mu L}\right)=\frac{cell\;count\times precision\;count\;beads\;volume}{precision\;count\;beads\;count\times cell\;volume}\times precision\;count\;beads\;concentration\;\left(\frac{beads}{\mu L}\right)$$

### Cell Viability Assays

Cell viability was assessed by culturing 100 × 10^3^ human total CD3^+^ T cells in unmodified HAGM cryogels, collagen, or media for 24, 48, and 72 h, after which the cells were harvested. Following 0.5 h of adherence of the cells to the 16 μL unmodified HAGM cryogels, an additional 180 μL of prewarmed medium was added to the cell-seeded gels, and they were incubated at 37 °C for the duration of the assay. Cells were retrieved from the HAGM cryogels as described above, and they were retrieved from collagen hydrogels by enzymatic digestion for 45 min at 37 °C with 1 mg/mL collagenase A (Roche). Cell viability was evaluated by flowcytometric analysis after staining eFluor780 (BD Pharmingen) and was compared to viability in medium and collagen hydrogels produced as described.^42^

### Injection Assays

For the injection assays with indium oxine, total human CD3^+^ T cells or murine (OT-I) CD8α^+^ T cells were isolated as described above. Isolated cells were dissolved in 500 μL of PBS to which 12 MBq of In-III-oxine (Curium Netherlands B.V.) was added. Cells were incubated for 15 min at RT after which cells were washed three times with 1 mL of fresh PBS. Following the In–III-oxine labeling, cells were added to the HAGM cryogels in 16 or 20 μL and adhered for 1–2 h. Collagen hydrogels of 1 mL were produced as described,^42^ and HAGM cryogels loaded with radioactive T cells were injected into the collagen hydrogels using 200 μL PBS and a 16G needle. Cryogels were retrieved from the collagen hydrogel at different time points, and radioactivity of the HAGM cryogels and collagen hydrogel was measured using an MCA (multichannel analyzer) with a well-type of sodium iodide (NaI) crystal.

For the injection experiment looking at the activation of the cells, total human CD3^+^ T cells were isolated and stained with CTV as described above. HAGM cryogels were loaded with 1 × 10^6^, 5 × 10^6^, or 10 × 10^6^ total human CD3^+^ T cells and injected in 2 mL of media in a 24-well plate. Following injection, the HAGM cryogels were either left in the well (injection together) or transferred to a new 24 well with 2 mL of media (Injection separate). Cells were retrieved from the scaffolds after 3 days, and CD25 expression and proliferation were assessed using flow cytometry analysis.

### Statistical Analysis

Data is expressed as mean ± SEM. Data was analyzed using GraphPad Prism 8.02 using the appropriate testing methods and normalization, as indicated in the figure legends. A *p*-value ≤0.05 was statistically significant. NS = nonsignificant, *p ≤ 0.05, **p ≤ 0.01, ***p ≤ 0.001, and ****p ≤ 0.0001.

## Results

### Preparation and Characterization of HAGM Cryogels

HAGM cryogels were prepared by polymerizing HAGM using a free-radical cross-linking process that was completed after 17 h at −20 °C.^59,60^ During this process, the HAGM polymers are cross-linked around ice crystals, which serve as natural porogens. The cooling rate^61^ and the size and structure of the ice crystals determine the porosity of the formed polymer network. After thawing, macroporous 3D HAGM cryogels with average homogeneous pore sizes of 54 ± 1.2 μm were obtained (Figure S1A,B). The porous network is highly interconnected (Figure S1C,D), which is beneficial, as it provides a large surface area for interaction with immune cells, facilitates cellular infiltration, and promotes diffusion of nutrients and chemical cues through the entire scaffold. The cryogels’ macroporous nature with high pore connectivity is one of their unique properties, making them closely resemble soft tissues.^62^ Moreover, the cryogels can be injected through conventional 16G hypodermic needles regaining their original structure (Figure S1E), which is in line with the reported mechanical stability and elasticity.^52,62^ The porous and injectable properties of HAGM cryogels make them ideally suited as a 3D environment for T-cell stimulation.

### Optimizing the HAGM Scaffold Design Parameters for Efficient Biomolecule Coupling

To be able to use HAGM cryogels as synthetic 3D T-cell-stimulatory immune niches, we devised a strategy to functionalize HAGM cryogels by covalently attaching various T-cell-stimulating biomolecules onto the polymeric walls.^54^ Attaching biomolecules to preformed cryogels rather than adding them before cryogelation^43–45,63^ is highly advantageous. This approach prevents biomolecules from being exposed to free radicals during cryopolymerization and undergoing freeze–thaw cycles, which can impair their bioactivity.^64^ Furthermore, this approach ensures that biomolecules are presented only externally on scaffold walls instead of being entrapped within the dense polymer network of the scaffold. To attach biomolecules for T-cell stimulation, we exploited the remaining carboxylic acids present in HAGM, which were not consumed through glycidyl methacrylation and reacted these with the primary amine of an azido-propylamine linker through EDC/NHS chemistry (Figure 1A). A copper-free click reaction was then used to conjugate dibenzocyclooctyne-amine (DBCO) functionalized, fluorophore-labeled biomolecules to the azide-containing cryogels.

**Figure 1:**
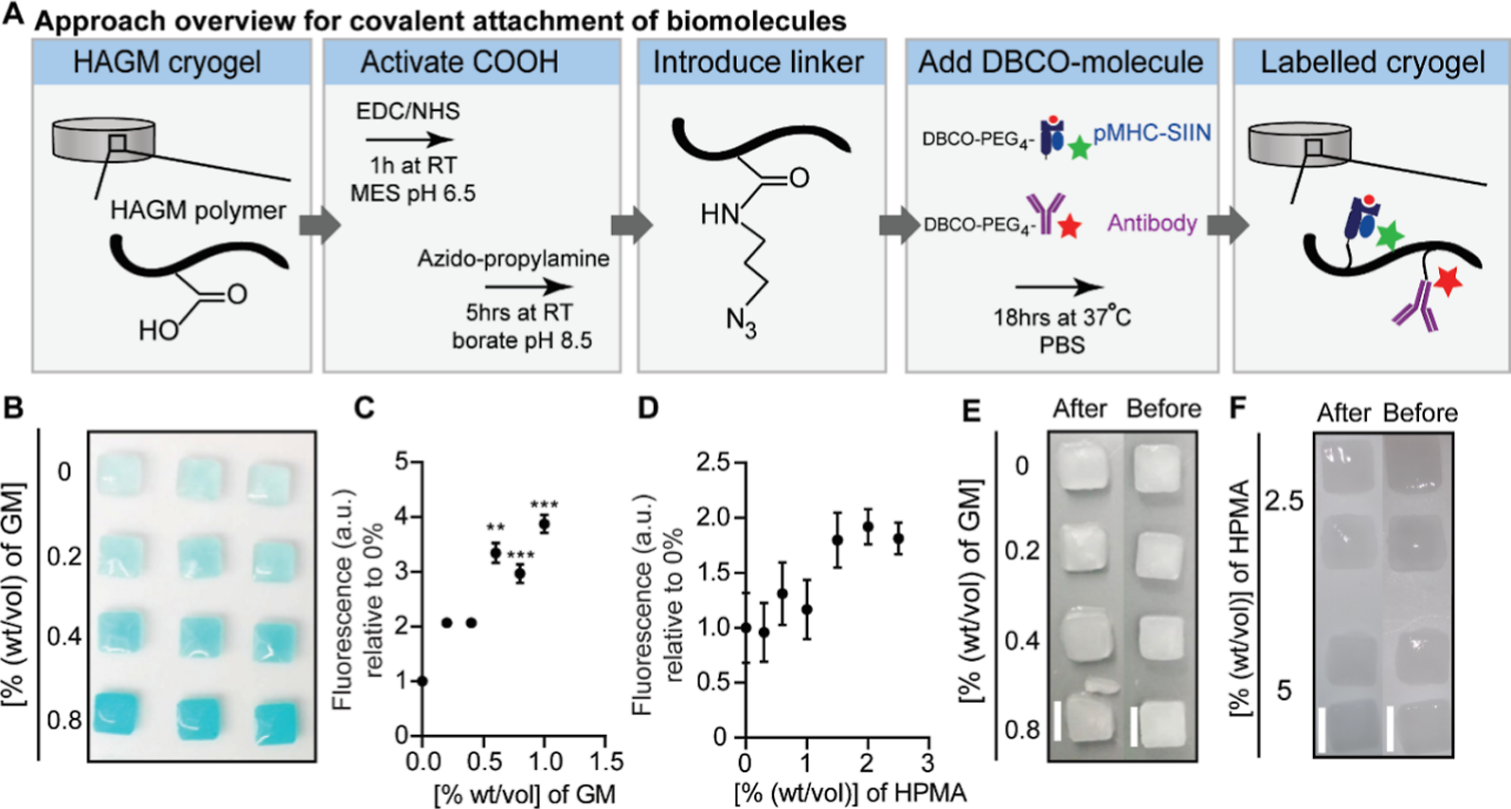
Importance of comonomers for the covalent attachment of biomolecules and integrity to HAGM cryogels. (A) Overview of the approach to covalently attach biomolecules (pMHC complexes and antibodies) to the polymer walls of preformed HAGM cryogels. RT (room temperature), colored stars indicate the fluorescent dye conjugated to the biomolecules for quantification of biomolecule conjugation to the scaffold. (B,C) Representative macroscopic image (B) and fluorescence quantification (C) of [3% (wt/vol)] cryogels made with increasing amounts of GM and labeled with an amine-Cy5 linker. (D) Fluorescence quantification of [3% (wt/vol)] HAGM cryogels made with increasing amounts of HPMA and labeled with an amine-Cy5 linker. (E) A representative image of the injectability of [3% (wt/vol)] HAGM cryogels with differing amounts of GM through a 16G needle. Scale bar equals 4 mm. (F) The injectability of [3% (wt/vol)] HAGM cryogels with 2.5 or 5 [% (wt/vol)] of HPMA through a 16G needle was tested. Scale bar equals 4 mm. (C,E) *n* = 3–9 in 1–3 independent experiments. Statistical significance was tested on log-transformed data comparing fluorescence to [0% (wt/vol)] GM or HPMA using a Kruskal–Wallis test with a Dunnett’s multiple comparisons correction.

After the synthesis of HAGM polymers, unreacted GM can remain in the final product. Free GM can act as a comonomer during cryopolymerization of HAGM cryogels and thereby influence the cross-linking process. To determine whether this free GM influenced the conjugation of biomolecules to the carboxylic acids present in HAGM, we used two different batches of HAGM, HAGM_LOW_ and HAGM_HIGH_. The HAGM_LOW_ batch contained negligible amounts of unreacted GM, and the HAGM_HIGH_ batch contained around 87% unreacted GM (Figure S2A,B). Importantly, we observed that the presence of unreacted GM was essential for the conjugation of biomolecules to the scaffold and the consequent activation of T cells (Figure S2C–F). We next set out to confirm our observations and investigate whether deliberate addition of GM to the HAGM_LOW_ batch could improve the biomolecule attachment to HAGM cryogels. We detected a dose-dependent increase of HAGM cryogel labeling upon addition of GM as a comonomer, with a 3.3× fold increased labeling with [0.6% (wt/vol)] GM (Figure 1B,C). We, however, observed that GM is also able to boost conjugation efficiency when carboxylic acids on HAGM are not activated with EDC/NHS (Figure S2G), which is likely due to the additional reactive (epoxide) group in GM. We therefore changed to *N*-(2-hydroxypropylmethacrylamide) (HPMA) as a comonomer to allow for more control and specificity over biomolecule conjugation. HPMA addition resulted in a dose-dependent increase of HAGM cryogel labeling, where we observed a 2-fold increase with [1.5% (w/v)] HPMA (Figure 1D). Importantly, the presence of the bio-orthogonal conjugation handle is crucial, since in the absence of the azido-propylamine linker, we only observed minimal background cryogel labeling with DBCO-Cy5 (Figure S2H). Interestingly, we observed that scaffold stability and deformability were slightly impaired when high amounts of GM were added (Figure 1E). In contrast, high amounts of HPMA did not reduce the scaffold stability but instead seemed to improve the injectability of the scaffolds (Figure 1F). For these reasons, we continued with addition of HPMA to HAGM batches with low amounts of free GM to enable efficient and specific conjugation of biomolecules to the scaffolds. In conclusion, the presence of comonomers during cryogelation is essential to facilitate covalent attachment of DBCO-functionalized biomolecules to preformed HAGM cryogels.

### Covalent Attachment of T-Cell-Stimulating Biomolecules to HAGM Cryogels

To create a scaffold capable of activating human polyclonal T cells, we applied the labeling strategy depicted in Figure 1A using DBCO-antihuman CD3 (αhCD3) and DBCO-αhCD28 mAbs. We added αhCD3 and αhCD28 mAbs in a ratio of 1:5, as higher amounts of αhCD28 have shown to improve T-cell activation.^65^ The fluorescently labeled mAbs bound in a homogeneous fashion throughout the scaffold and localized with the wall structures of the cryogel macroporous network (Figure 2A). The fluorescent signal for αhCD3 and αhCD28 colocalized to a high degree, suggesting that these signals are in close proximity to each other and could potentially be copresented to T cells. Quantification of the fluorescence demonstrated dose-dependent attachment of αhCD3 and αhCD28 mAbs to the cryogels, with 8-fold more αhCD3 and 16-fold more αhCD28 bound when the amount of DBCO-mAb added was increased to 4 and 20 μg, respectively (Figure 2B). We observed an incorporation efficiency for αhCD3 between 34% and 42% of the total amount of αhCD3 that was added. The incorporation efficiency for αhCD28 varied between 22% and 37% (Figure 2C). This resulted in HAGM cryogels functionalized with 0.2 μg of αhCD3 and 0.5 μg of αhCD28, 0.9 μg of αhCD3 and 3.7 μg of αhCD28, and 1.6 μg of αhCD3 and 8.4 μg of αhCD28, respectively.

**Figure 2:**
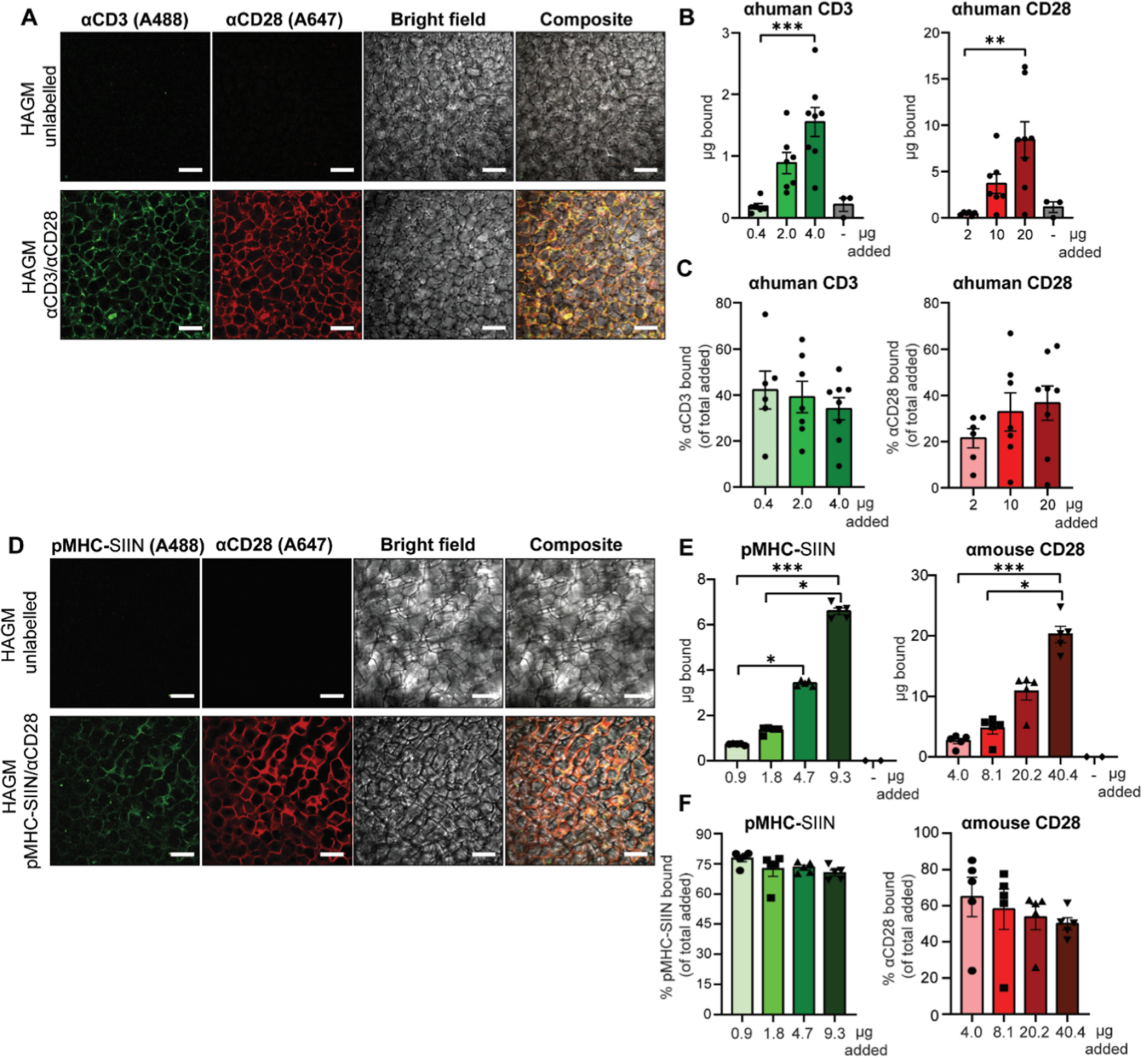
Strategy to functionalize HAGM cryogels with T cell-stimulating signals. (A) Representative confocal microscopic images of [4% (wt/vol)] low molecular weight (LMW) HAGM cryogels labeled with 4.0 μg human αCD3-A488 and 20 μg human αCD28-A647. Scale bar equals 100 μm. (B) Fluorescence quantification of HAGM cryogels labeled with increasing concentrations of human αCD3-A488 and human αCD28-A647 antibodies. (C) Labeling efficiency of human αCD3-A488 and human αCD28-A647 antibodies. Percentage of total added mAb that has bound to the HAGM cryogels. (D) Representative confocal microscopic images of [4% (wt/vol)] HAGM cryogels functionalized with 9.3 μg mouse pMHC-A488 (H-2K^b^ SIINFEKL) and 40.4 μg mouse αCD28-A647. Scale bar equals 100 μm. (E) Fluorescence quantification of HAGM cryogels labeled with increasing concentrations of mouse pMHC and mouse αCD28-A647 antibodies. (F) Labeling efficiency of mouse pMHC and αmCD28-A647 antibodies. Percentage of added mAb that have bound to the HAGM cryogels. (B,C) *n* = 6–10 in 3–5 independent experiments. (E,F) *n* = 5 in 3 independent experiments. (B,E) Statistical significance of labeling was evaluated by a Kruskal–Wallis test with a Dunn’s correction.

To activate mouse T cells in an antigen-specific manner, cryogels were functionalized with DBCO-functionalized MHC class I H-2K^b^ complexes loaded with ovalbumin-derived peptide SIINFEKL (pMHC-SIIN) in combination with DBCO-antimurine CD28 (αmCD28) mAbs. Similar to the human cryogel design, we added more mαCD28 mAb to the cryogels compared to the pMHC-SIIN with a ratio of 1:2 (pMHC-SIIN/αmCD28).^65^ Both the pMHC-SIIN and the αmCD28 localized in a network-specific manner and in a homogeneous fashion throughout the scaffold (Figure 2D). Moreover, both the pMHC-SIIN and the αmCD28 bound in a linker-specific manner, as we observed that the cryogels without the azide linker displayed only a low background signal (Figure S2I,J). Quantification of the fluorescence signals in the cryogels for both biomolecules indicated that there was dose-dependent labeling, as we observed significantly more pMHC-SIIN signal with the addition of 4.7 or 9.3 μg compared to 0.9 μg pMHC-SIIN and significantly more αmCD28 with the addition of 40 μg compared to the addition of 4.0 or 8.1 μg αmCD28 during labeling (Figure 2E). The labeling efficiency of pMHC-SIIN was similar for the different starting conditions, ranging from 70% to 78% (Figure 2F). For αmCD28, the labeling efficiency was slightly lower, between 50% and 65% (Figure 2F). Overall, these results show that the conjugation of T-cell-activating biomolecules onto HAGM cryogels is effective and that the labeling strategy is highly modular.

### HAGM Cryogels Are Potent T Cell-Stimulating Immune Niches

Next, we established whether the 3D HAGM cryogels could support T cell survival, expansion, and activation. To this end, we isolated total human CD3^+^ T cells from healthy human donors and compared the viability in HAGM scaffolds with traditional T cell cultures in medium (2D) and 3D collagen gels. Viability after 24, 48, and 72 h was similar for all conditions (Figure S3A), indicating that HAGM cryogels accommodate T-cell survival. To study the impact of ligand density on T-cell activation in our scaffold system, we generated HAGM scaffolds with increasing amounts of T-cell-activating αhCD3/αhCD28 mAb (HAGM + mAb). HAGM cryogels without mAbs did not induce T-cell proliferation or cytokine production (Figure 3A–D). In contrast, HAGM + mAb cryogels induced antibody-dose-dependent T-cell activation. HAGM + mAb with high αhCD3 and αhCD28 induced significantly higher levels of CD8^+^ T-cell proliferation compared to blank HAGM cryogels or the HAGM + mAb labeled with low αhCD3 and αhCD28 amounts (Figure 3B). HAGM + mAb additionally increased IL-2, IFNγ, and TNFα production compared to the blank HAGM cryogels (Figure 3C). Thus, HAGM cryogels labeled with mAb can induce human polyclonal T-cell proliferation and cytokine production.

**Figure 3:**
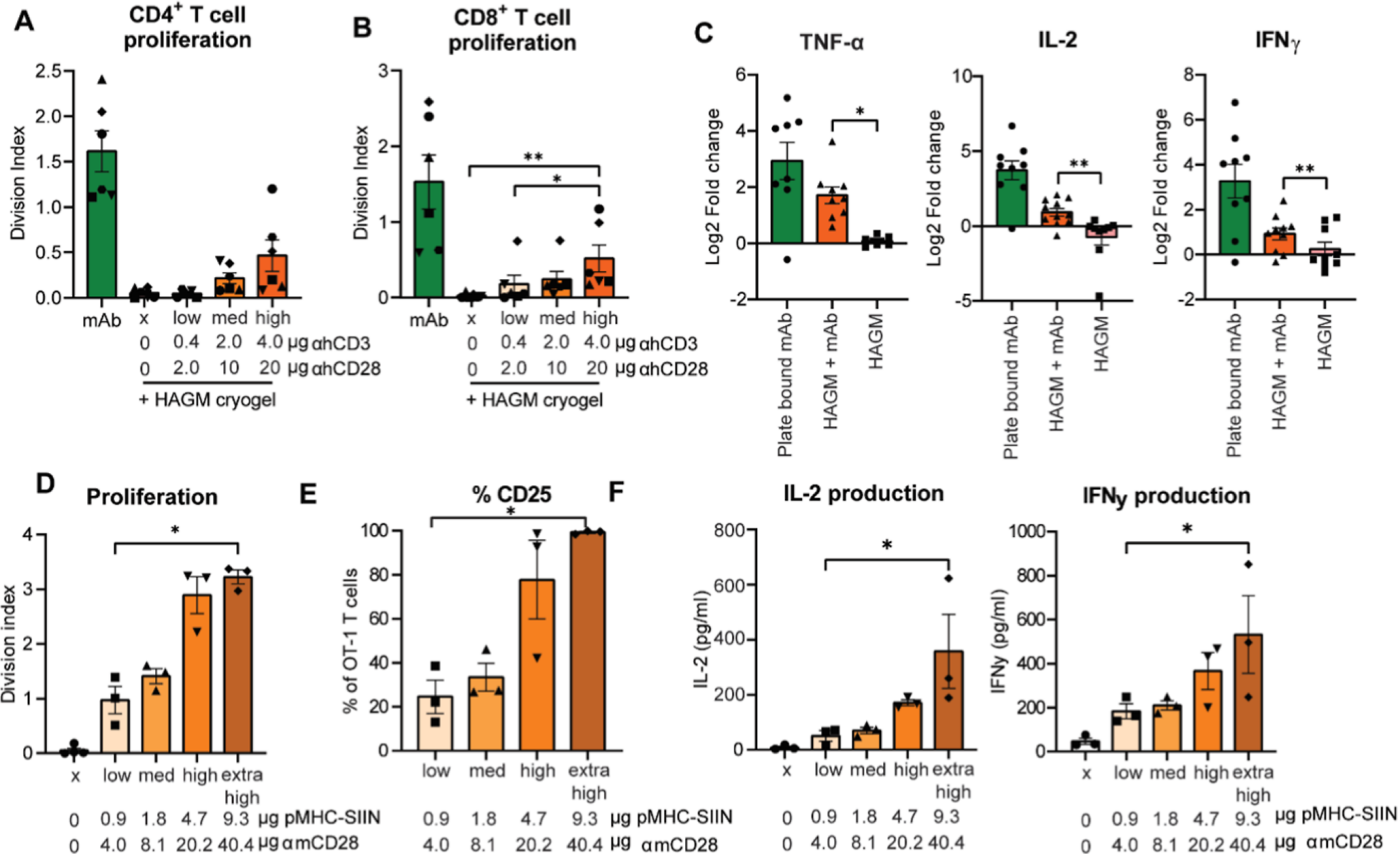
HAGM cryogels labeled with T-cell stimulatory signals induce T-cell expansion and cytokine secretion. Proliferation of human (A) CD4^+^ and (B) CD8^+^ T cells upon 96 h exposure to blank HAGM cryogels or cryogels labeled with mAb. Division index was determined by flow cytometric analysis, measuring Cell Trace Violet dilution. (C) Cytokine production by human T cells (IFNγ, IL-2, and TNF-α) after 24 h measured by ELISA. (D) Division index after 72 h stimulation of murine OT-I CD8^+^ T cells within HAGM cryogels labeled with increasing amounts of pMHC-SIINFEKL and αmCD28. (E) Percentage of OT-I T cells expressing the activation marker CD25, 72 h following stimulation. (F) Cytokine production by OT-I T cells (IL-2 and IFNγ) production measured by ELISA 24 h following stimulation. (A,B) *n* = 6 in 3 independent experiments, each symbol represents an individual healthy donor. Log normalized data was analyzed by a (A) mixed-effects analysis with a Sidak correction or by a (B) Friedman test with Dunn’s multiple comparison correction. (C) *n* = 6–11 in 7 independent experiments. Raw data was analyzed with a paired *t*-test (TNFα) or by a Wilcoxon test (IL-2 and IFNγ) comparing HAGM + mAb to HAGM. (D–F) *n* = 3–4 in 3–4 independent experiments. Data was analyzed with a Friedmann’s test with a Dunn’s multiple comparison correction, comparing every condition to the 0.9 μg pMHC-SIINFEKL and αmCD28 μg 4.0 condition.

Next, we investigated the potential of our functionalized HAGM niches to activate antigen-specific murine T cells using pMHC-SIIN complexes in combination with αmCD28. This approach is particularly important to support induction of antigen-specific or neoantigen-specific T-cell responses in vivo or to expand antigen-specific T cells from mixed lymphocyte populations, e.g., tumor-infiltrating lymphocytes (TILs). To this end, we conjugated pMHC-SIIN and αmCD28 to the scaffolds and added CD8^+^ T cells from the OT-I transgenic mice. These OT-I T cells express a TCR that specifically recognizes the SIINFEKL peptide in the context of MHC class I H-2K^b^. After stimulation for 72 h in the HAGM cryogels, OT-I T-cell proliferation increased in a distinct dose-dependent manner, with significantly more proliferation for HAGM cryogels labeled with higher levels of pMHC-SIIN and αmCD28 (division index of 3.2 ± 0.1) compared to HAGM cryogels labeled with low levels of pMHC-SIIN and αmCD28 (division index of 0.98 ± 0.25, Figure 3D). A similar dose-dependent increase was observed in the percentage of OT-I T cells expressing the activation marker CD25 (Figure 3E) and their production of the cytokines IL-2 and IFNγ (Figure 3F). These data confirm that HAGM cryogels functionalized with T-cell stimulatory cues can activate and expand both human and murine T cells either in a polyclonal or in an antigen-specific manner.

### 3D HAGM Cryogels Induce Similar Levels of T-Cell Expansion and Memory Phenotypes Compared to 2D Cultures

Next, we used HAGM cryogels presenting at least 1 μg of αhCD3 and 5 μg of αhCD28 to determine their T-cell expansion abilities during long-term culture (up to 14 days). We observed significantly higher human T-cell expansion in our HAGM + mAb cryogels by day 14 compared to media alone or blank HAGM cryogels but no significant differences compared to 2D plate bound cultures (Figure 4A). Interestingly, HAGM cryogels induced equal levels of CD4^+^ and CD8^+^ T-cell expansion, while culture of the T cells in the 2D environment with plate bound mAb resulted in twice the amount of CD4^+^ T cells compared to CD8^+^ T cells (Figure 4A). When retrieving the T cells from the scaffolds, a distinction can be made between cells retrieved from inside the HAGM cryogels (HAGM + mAb in) and from the media surrounding the scaffolds (HAGM + mAb out), which contain T cells that have moved out of the scaffold. Interestingly, an increased CD4^+^ T-cell expansion was measured outside the HAGM cryogels, albeit without statistical significance, whereas the cell counts of CD8^+^ T cells inside and outside the HAGM cryogels were similar for all time points (Figure S3B). Overall, this indicates that the HAGM cryogels support the expansion of T cells inside the 3D immune niche and in the environment around the scaffold over prolonged periods of time.

**Figure 4:**
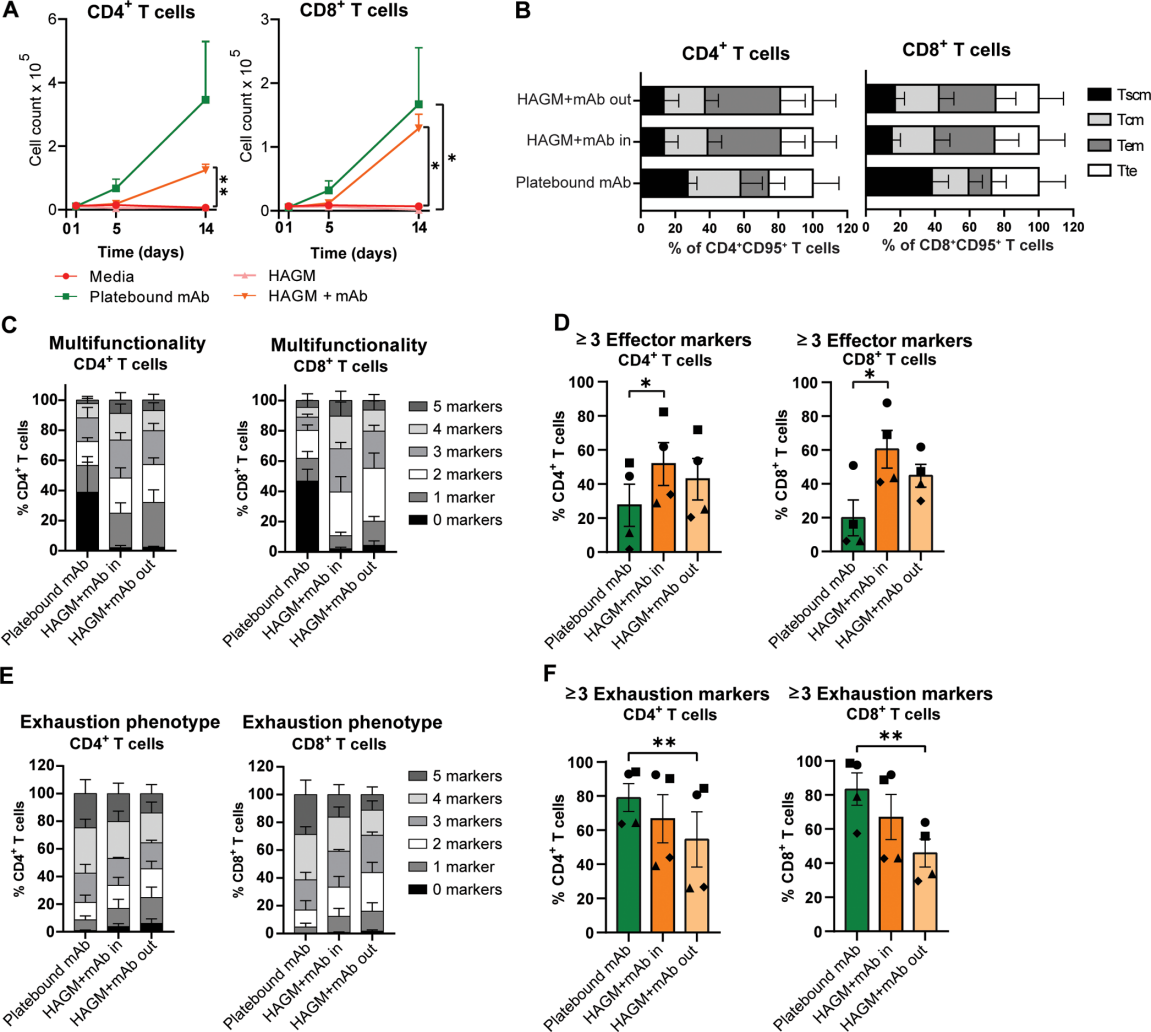
HAGM cryogels induce a more multifunctional phenotype and a less exhausted T cell phenotype. (A) CD4^+^ and CD8^+^ T-cell expansion during 14 days of culture with media, plate bound mAb, blank HAGM cryogel, or HAGM + mAb cryogel. (B) Memory phenotype of CD4^+^ T-cells and CD8^+^ T-cell differentiation following the linear T-cell differentiation model, according to which cells differentiate from Tscm (CD95^+^CCR7^+^CD45RA^+^) > Tcm (CD95^+^CCR7^+^CD45RA^–^) > Tem (CD95^+^CCR7^–^CD45RA^–^) > Tte (CD95^+^CCR7^–^CD45RA^+^). (C) Percentage of CD4^+^ and CD8^+^ T cells expressing between 0 and 5 effector molecules (perforin, GrzB, TNF-α, IL-2, IFNγ) on day 5 of stimulation. (D) Percentage of multifunctional CD4^+^ and CD8^+^ T cells expressing 3 or more effector molecules following 5 days of stimulation. (E) Percentage of CD4^+^ and CD8^+^ T cells expressing between 0 and 5 exhaustion markers (CD39, CTLA-4, PD-1, TIGIT, TIM-3) by day 14 of culture. (F) Percentage of CD4^+^ and CD8^+^ T cells expressing 3 or more exhaustion markers following 14 days of stimulation. (A) *n* = 6 in 4 independent experiments. Data was analyzed by a Friedman test with a Dunn’s correction. Comparing media vs plate bound mAb, HAGM vs HAGM + mAb, and plate bound mAb vs HAGM + mAb. (B–F) *n* = 4 in 2–3 independent experiments. (D,F) Each symbol represents a healthy individual donor. Data was logit transformed and analyzed with a Friedman test with a Dunn’s multiple comparison correction, comparing the mean of each column with the mean of every other column.

In addition to the expansion of T cells, we also established the memory phenotype of the T cells in the 3D immune niches. T-cell memory phenotypes can be subdivided into five populations using the linear T-cell differentiation model: naïve, stem cell memory (Tscm), central memory (Tcm), effector memory (Tem), and terminal effector (Tte).^66^ Considering ACT strategies, it has been noted that generating T cells with an earlier memory phenotype (Tscm/Tcm) is preferential, as these T cells exhibit superior survival, proliferation, and sustained effector function in vivo.^67–70^ At the start of the culture, 74.5% of the CD4^+^ and 80.3% of the CD8^+^ T cells were CD95^+^, which is indicative of a memory phenotype. By day 5, all CD4^+^ and CD8^+^ T cells had a memory phenotype (99%), which were mostly early memory T cells (Tscm or Tcm) (Figure S3C,D). By day 14, this shifted toward a more effector memory phenotype for the CD4^+^ T cells cultured in the HAGM + mAb cryogels (HAGM + mAb in), with 61.8% depicting a Tem or Tte phenotype (Figure 4B). The CD8^+^ T cells displayed all four memory phenotypes to a similar extent by day 14 (Figure 4B). For both CD4^+^ and CD8^+^ T cells, there was no difference in the memory phenotype of cells harvested from inside (HAGM + mAb in) or outside the cryogels (HAGM + mAb out) (Figure 4B). When compared to the plate bound mAb culture, no significant differences in memory phenotype were observed; however, the plate bound mAb condition has a tendency to favor the Tscm and Tte memory phenotypes in both CD4^+^ and CD8^+^ T cells, which we do not observe for the HAGM + mAb conditions. In conclusion, culturing T cells in 2D media conditions or in 3D HAGM cryogels affect the T-cell memory phenotype in a similar way.

### 2D Cultures Result in More Exhausted T Cells with a Less Multifunctional Phenotype Compared to More Natural 3D Immune Niches

Next, we evaluated the multifunctionality of T cells on day 5 of the culture in the 3D HAGM cryogels with αhCD3 and αhCD28. A multifunctional profile is critical for the antitumor function of T cells.^71^ T cells are defined as being multifunctional when expressing at least two effector markers, which include cytokines, chemokines, degranulation markers, and cytotoxic molecules.^72^ We investigated the coexpression of IFNγ, TNF-α, IL-2, perforin, and granzyme B (GrzB). A larger percentage of the T cells stimulated with the plate bound mAb expressed no effector markers compared to the T cells cultured in the HAGM cryogels (Figure 4C). Moreover, T cells retrieved from within the HAGM cryogels (HAGM + mAb in) showed significantly higher levels of multifunctionality compared to plate bound mAb activation, resulting in twice the number of multifunctional CD4^+^ T cells and three times more multifunctional CD8^+^ T cells (Figure 4D). Importantly, T cells retrieved from the media surrounding the scaffold (HAGM + mAb out) also displayed a multifunctional phenotype (Figure 4C). The majority of the CD4^+^ T cells expressed a combination of GrzB, IL-2 and TNF-α, whereas the majority of the CD8^+^ T cells expressed a combination of GrzB, perforin, and TNF-α (Figure S3E). The continued activation outside the scaffold might be due to binding of mAb to the outer surfaces of the scaffold and thus providing T cells outside the scaffold with stimulatory signals. Alternatively, it could be indicative of durable T cell activation inside the scaffolds, which continues after T cells exit the activating 3D environment, underlining the importance of the context in which T cells encounter biomolecules.

Continuous exposure of T cells to stimulation can cause dysfunctional T cells with limited capacity to produce cytokines and ability to kill target cells.^73^ This exhausted state is characterized by sustained expression of multiple inhibitory receptors.^73^ Both in the plate bound mAb conditions and in the HAGM + mAb conditions, stimulatory mAbs are continuously present. We were therefore interested in determining human T-cell exhaustion following prolonged cell culture. To this end, we investigated the coexpression of 5 distinct coinhibitory receptors: programmed death 1 (PD-1), CTLA-4, T-cell immunoglobulin and mucin-domain containing-3 (TIM-3), T-cell immunoreceptor with Ig and ITIM domains (TIGIT), and CD39. Individual expression of these markers can also be indicative of reactivated T cells; we therefore looked into cells coexpressing at least 3 of the measured markers at day 14 of culture to determine the exhaustion state. There was a trend toward fewer exhausted T cells when cultured in the 3D HAGM cryogels (HAGM + mAb in) compared to the 2D culture with plate bound mAbs (Figures 4E and S3F). T cells retrieved from outside the 3D HAGM cryogels (HAGM + mAb out) showed significantly lower percentages of multiple exhaustion markers compared to the 2D plate bound culture, around 30% less exhausted CD4^+^ T cells and 45% less exhausted CD8^+^ T cells (Figure 4F). When looking at the individual contribution of each exhaustion marker, it appears that TIGIT, PD-1, and CTLA-4 are mainly expressed in combination with CD39 or CD39 and TIM-3 as depicted by the pie charts (Figure S3F). Altogether, these results indicate that our 3D HAGM scaffold provides a stimulatory environment for T cells that induces high levels of multifunctional T cells with lower levels of exhaustion marker expression compared to 2D cultures.

### HAGM Cryogels as Injectable Stimulatory Immune Niches

The possibility for in vivo T cell activation and expansion could circumvent the challenges of the laborious ex vivo expansion process in current ACT strategies. For this purpose, different scaffold designs have been developed for ACT of chimeric antigen-receptor (CAR) T cells.^40,41,74–78^ A key benefit of applying HAGM scaffolds for T-cell stimulation compared to other biomaterial-based scaffolds is their biodegradability and their ability to be delivered preformed through needle-mediated injection owing to their deformability. We therefore studied the impact of injection of T-cell-loaded HAGM cryogels on T cell viability and retention. To this end, human T cells were added to the HAGM scaffolds in low volumes (16 or 20 μL) and adhered for 1 or 2 h prior to injection through a 16G needle. Viability of human T cells was not affected by either adherence time or adherence volume prior to injection (Figure S4A). We did observe a slight decrease in viability upon injection (76.5 ± 4.4%) compared to no injection (94.9 ± 0.9%) (Figure S4B). To study the retention of the T cells following injection, we radioactively labeled human total CD3^+^ T cells with indium-111 oxyquinoline. The retention of T cells within the HAGM cryogels following injection varied between 45 and 56% and was not impacted by the number of T cells that adhered to the scaffolds, the adherence volume, or the presence of stimulatory antibodies (Figures 5A and S4C). Following injection, T cells were able to migrate through the macroporous scaffold network and move out of the scaffolds, as evidenced by an increase in radioactivity in the surrounding collagen matrix over the course of 3 days (Figure 5B).

**Figure 5:**
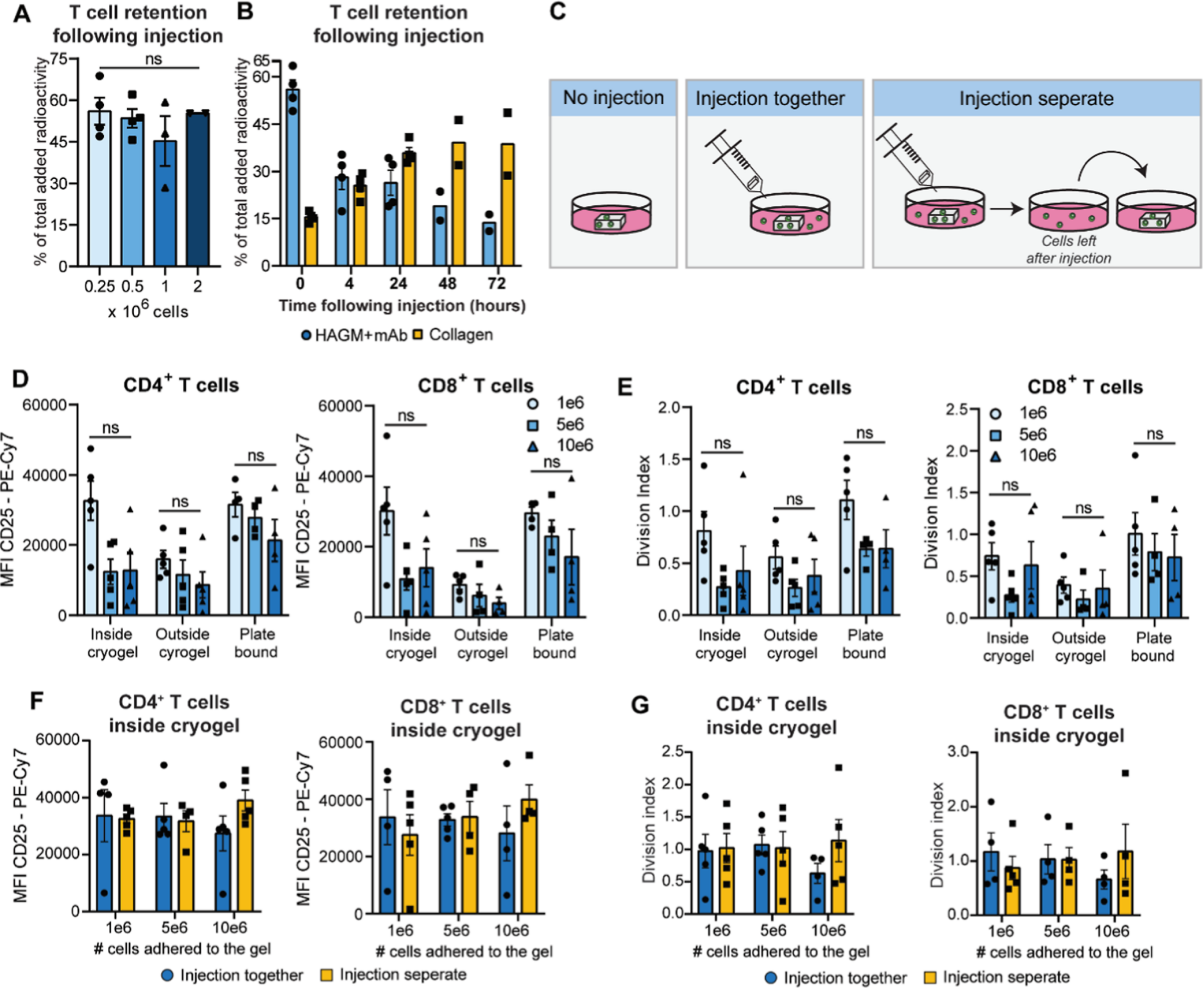
Human T-cell retention and activation in HAGM cryogels following injection. (A) Human total CD3^+^ T cells were radioactively labeled with indium oxine, adhered to the HAGM cryogels, and injected into collagen matrices. Percentage of human total CD3^+^T-cell retention in HAGM cryogels with scaffolds functionalized with αCD3 + αCD28 Ab following injection of 0.25 × 10^6^, 0.5 × 10^6^, 1 × 10^6^, or 2 × 10^6^ T cells. (B) Percent of 0.5 × 10^6^ human total CD3^+^T-cell retention in HAGM cryogels with αCD3 + αCD28 Ab and localization in collagen matrix following injection. T-cell retention was measured up to 72 h following injection. (C) Schematic overview of (1) HAGM scaffolds with T cells not injected, (2) HAGM scaffolds with T cells that were injected and cultured together for 3 days, and (3) HAGM scaffolds with T cells that were injected and cultured in separate wells following injection. (D) CD25 expression and (E) division index of increasing numbers of human CD4^+^ T cells and CD8^+^ T cells cultured in HAGM scaffolds + mAb for 3 days. (F) CD25 expression and (G) division index of CD4^+^ and CD8^+^ human T cells retrieved from inside the HAGM scaffolds on day 3 that were injected and either cultured together or separate. (A,B) *n* = 4 in 2 independent experiments. (D–G) *n* = 5 in 4 independent experiments. (A) Data evaluated with one-way ANOVA with Tukey correction. (D-G) Data evaluated with mixed-effects analysis with a Tukey correction.

We then investigated murine T-cell retention in HAGM scaffolds following injection into a collagen matrix. We found that 43.2 ± 2.6% of both wildtype CD8^+^ T cells and antigen-specific OT-I CD8^+^ T cells were retained within the scaffolds and were able to migrate out of the scaffolds, irrespective of the presence of stimulatory antibodies (Figure S4D,E). Together, these data suggest that the HAGM cryogels can support ACT of (antigen-specific) T cells and that 40 to 60% of T cells are retained in the scaffold directly following injection. Moreover, the T cells can leave the scaffold over time, which is imperative for their functionality in vivo.

Next, we investigated whether the injection itself had an impact on T cell activation and proliferation (Figure 5C). We first assessed that the amount of T cells loaded into the HAGM scaffolds prior to injection did not influence their activation or proliferation (Figure 5D,E). We then found that T cells are activated and proliferate to the same extent inside and outside of the scaffolds irrespective of whether the HAGM scaffold was injected (Figures 5F–G and S4F,G). Interestingly, the T cells that had only been in contact with the scaffold during the adhesion time (30 min) and were cultured in a separate well following injection had also upregulated the activation marker CD25 and had divided to the same extent over the course of 3 days (Figure S4H,I).

Overall, these findings underline the potential of HAGM scaffolds to support T-cell activation during ACT. We showed that the injection of up to 10 million T cells loaded into our HAGM scaffolds resulted in potent T-cell activation both inside and outside the scaffold. Interestingly, T cells that were directly flushed out of the scaffold upon injection and therefore did not have the possibility to interact with the scaffold at a later time point preserved proliferative capacity, indicating that the short-term initial exposure of 30 min to the activating signals in the 3D synthetic immune niche was sufficient to induce T-cell activation.

## Discussion

The expansion of T cells ex vivo is one of the more challenging steps in the current ACT therapeutic strategies, as this process is not only laborious but can also negatively impact T-cell viability and functionality.^18^ The possibility of in vivo activation and expansion of immune cells could circumvent many of these drawbacks. Recently, different scaffold designs have been tested to explore their potential in aiding the ACT of CAR-T cells.^40,41,74–80^ Here, we demonstrate a strategy to functionalize HAGM cryogels for the activation and expansion of T cells. An advantage of HAGM scaffolds for T-cell stimulation compared to other biomaterial-based scaffolds is their ability to be delivered preformed through needle-mediated injection owing to their favorable mechanical properties. The mechanical stability and deformability of HAGM cryogels have been extensively studied before, where it was found that careful balancing of ice nucleation and the rate of free radical polymerization during cryogelation is pivotal to create mechanically robust and defined scaffolds with sufficient pore connectivity, which can withstand repeated deformation of over 90% strain level.^62,81^ As such, these preformed scaffolds can be injected while retaining their shape and mechanical robustness as they release water when they flow through the needle and reabsorb water once they have passed the needle to regain their original shape and size. Injectable scaffolds circumvent the risk caused by open surgery otherwise needed for scaffold implantation. Moreover, the scaffold can function as a protective environment during injection, thereby preventing cell damage due to shear forces and ensure local and concise delivery of cells for ACT purposes.^82–84^ Additionally, our preformed injectable HAGM cryogel scaffolds do not rely on difficult-to-control in situ gelling conditions required for most injectable hydrogel-based scaffolds.^39,49,50,79,80,85^ In situ gelation of hydrogels can result in loss of cargo, poorly defined macrostructures and leakage of liquid precursors from the injection site, which can result in toxicity and limited control over the scaffold structure and location.^62,81,86^

In contrast to many hydrogels and other cryogel scaffolds, the HAGM scaffolds are manufactured using hyaluronic acid, which is a naturally occurring component of the extracellular matrix, making it biodegradable. Other groups have shown promising results using nonbiodegradable alginate cryogels to create 3D scaffolds for the activation of T cells.^41,43–45,87^ Our HAGM design builds on these studies to create a 3D scaffold that can be degraded over time following injection.

In order to functionalize HAGM scaffolds to enable the activation of T cells, we devised a strategy to conjugate T-cell activating biomolecules to the preformed scaffold walls. We have shown that the presence of comonomers during cryogelation (GM or HPMA) is essential to facilitate the covalent attachment of DBCO-functionalized biomolecules to the HAGM cryogels. We hypothesize that these comonomers act as additional linkers between the HAGM monomers, which provides more space and flexibility for the EDC/NHS reaction to utilize available COOH groups that protrude from the individual polymers in the scaffold walls. Importantly, by adopting this postcryogelation labeling strategy, we can ensure bioactivity of the biomolecules by preventing exposure of biomolecules to free radicals during cryogelation and avoiding freeze–thaw cycles.^64^ This strategy also guarantees that biomolecules are presented only externally on the walls of the scaffold and are thus available to the T cells. Importantly we observed continued T-cell activation outside the scaffold, which might be due to mAb binding to the outer surfaces of the scaffold. Alternatively, it could be indicative of durable T cell activation inside the scaffolds, which continues after T cells exit the activating 3D environment, underlining the importance of the context in which T cells encounter biomolecules.

In addition to the T-cell-activating biomolecules used in the current design, the modular character and easy adaptable nature of the scaffold allow for the incorporation of a wide variety of other (immunomodulating) molecules. For instance, other costimulatory signals could be used to boost and control T-cell function and fate, e.g., agonistic CD2, 4-1BB, or OX-40 antibodies.^88–90^ When antigen-specific T-cell expansion is necessary, a diverse repertoire of pMHC complexes would provide the opportunity to generate T-cell responses specific to a broad repertoire of tumor-associated antigens or neoantigens. Alternatively, we propose that the conjugation strategy demonstrated here could be useful in other fields to present biomolecules in a 3D environment to regulate cellular activation and differentiation. The context in which these signals are presented can be tuned by varying the scaffold macrostructure,^91,92^ introducing biomolecules through different chemistries or as soluble cues, or influencing the rate of cryogel degradation by adjusting the cross-link density or introducing cleavable linkers. In addition to the versatility that the scaffold design and conjugation strategy can offer, we envision that functionalized HAGM cryogels may also be applied to stimulate various types of (immune) cells. In the future, it would be particularly interesting to study their potential for the specific stimulation and expansion of CAR T cells or T cells genetically engineered to express certain T cell receptors, as the HAGM scaffolds might provide a dedicated niche for improved survival and functionality of these engineered T cells.

The scaffold design used here provided T cells with signals 1 and 2 (TCR stimulation and costimulation), while signal 3 (IL-2) was provided as a soluble factor in the media. Incorporation of cytokines into scaffold designs, such as IL-15, IL-15 super agonist, or IL-2, has shown to improve the in vivo antitumor response of T cells.^40,41,74,76–78^ Therefore, next steps in the development of the HAGM scaffold design could be the incorporation of cytokines, either by adding them prior to the cryogelation to encapsulate them in the scaffold walls or by modification of the scaffold with the glycosaminoglycan heparin to provide a cytokine binding moiety.^93^ The scaffold design could be further optimized by the addition of cell-adhesive ligands, such as cyclic RGD, to enhance cell attachment to the HAGM scaffold, as these signals have shown to improve migration and viability of T cells in vivo for several other scaffold designs.^40,94–96^

The use of 3D scaffolds for the stimulation of T cells has been studied more frequently as a means to improve the T cell expansion rates and functionality. For example, scaffolds based on mesoporous silica rods that present T cell-stimulation cues were shown to enhance the expansion of T cells compared to 2D cultures with αCD3/αCD28 Dynabeads.^37^ In our study, however, we did not observe increased expansion of T cells compared to 2D plate bound mAb cultures. More importantly, we did observe increased levels of T cell multifunctionality and lower levels of T cell exhaustion with our 3D scaffold systems compared to those of the 2D culture. Additionally, the HAGM scaffolds resulted in a balanced activation of both CD4^+^ and CD8^+^ T cells. In contrast, mesoporous silica rods showed a substantial CD8-bias, whereas the 2D culture with either plate bound mAb or with αCD3/αCD28 Dynabeads promoted a CD4^+^ T cell bias.^37^ It has been suggested recently that CAR-T cell products which harbor similar levels of CD4^+^ and CD8^+^ T cells are preferential for clinical use.^69,70,97^ The functionalized HAGM scaffolds are thus capable of robustly expanding functional T cells with a favorable CD4^+^:CD8^+^ balance, multifunctional profile, and limited exhaustion.

## Conclusion

The interest in 3D scaffolds for the activation, expansion, and regeneration of T cells has increased enormously in recent years. In this study, we devised a modular approach to functionalize HAGM-based scaffolds to create synthetic immune niches for antigen-specific and polyclonal T-cell activation. We have developed an efficient conjugation strategy to covalently attach different biomolecules with a relatively high yield to the 3D HAGM cryogels after cryogelation. By labeling the scaffold postcryogelation, we preserve the bioactivity of the biomolecules by preventing their exposure to free radicals during cryogelation and by avoiding freeze–thawing cycles. The functionalized scaffolds provide T cells with a 3D synthetic immune niche, in which they become activated. Importantly, T cells expanded rapidly, displayed a multifunctional phenotype, and demonstrated a less exhausted phenotype, even after being moved out the 3D scaffold environment, compared to T cells stimulated with plate bound mAb in a 2D environment. Our modular cryogel-based scaffold can be easily amended and can therefore be a valuable tool in the development of new local biomaterial-based cancer immunotherapies.

## Supplementary Material



## Notes

Characterization of structure, pore size, connectivity, and injectability of HAGM gels with confocal imaging and scanning electron microscopy; ^1^H NMR spectra of HAGM polymers; confocal imaging and fluorescence quantification of human-αCD3, pMHC-SIIN, and mouse αCD28 in HAGM gels with varying amounts of comonomers; T cell proliferation and IFNy production in HAGM gels with varying amounts of comonomers; human T cell viability, count, and memory phenotype in HAGM gels; retention of human T cells in HAGM gels following injection; antibodies and gating strategy used for flowcytometric determination of the memory, effector, and exhaustion phenotype of human T cells; and protein sequence of pMHC-SIIN (PDF)

## ¶

Department of Immunology, Leiden University Medical Center, Leiden, The Netherlands
