# Supplementary material for: Biomolecule Conjugation Strategy for HAGM Cryogels to Create 3D Immune Niches that Induce Multifunctional T Cells
Source: ACS Biomater Sci Eng. 2025 Jul 19;11(8):4773–87. doi: 10.1021/acsbiomaterials.5c00134 (PMC12344646; doi:10.1021/acsbiomaterials.5c00134)
Supplement: Supplementary file 1 [file ab5c00134_si_001.pdf]

Biomolecule conjugation strategy for HAGM cryogels to create 3D immune niches that induce multifunctional T cells

*Marjolein Schluck[#,1,2], Jorieke Weiden[#*1,2,3], Roel Hammink[1,2], Lea Weiss[1,2,3], M. Eloisa Vega Quiroz[1], Maren Pfirrmann[1], Laia Junquera Guinovart[1], Vincent van der Steen[1], Chadia Archidi[1], Leanne H. Minall[1], René Classens[1], Mahboobeh Rezaeeyazdi[4], Thibault Colombani[4], Sidi A. Bencherif[4,5], Carl G. Figdor[1,2.3], Martijn Verdoes[*1,3,6]*

[1]Department of Medical BioSciences, Radboud University Medical Center, Nijmegen, The Netherlands.
[2]Oncode Institute, The Netherlands.
[3]Institute for Chemical Immunology, The Netherlands.
[4]Department of Chemical Engineering, Northeastern University, Boston, MA, 02115 USA
[5]University Rouen Normandie, CNRS, PBS UMR 6270, F-76000, Rouen, France
[6]Current address: Department of Immunology, Leiden University Medical Center, Leiden, The Netherlands.

Supplemental Information

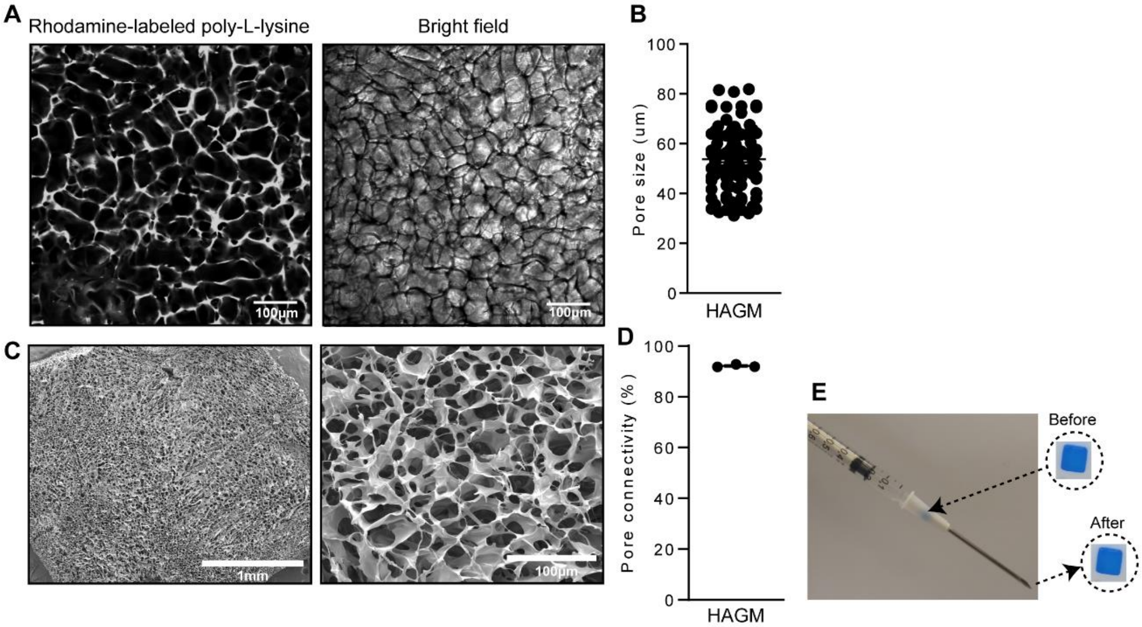

Supplemental Figure 1. HAGM cryogels are macroporous with interconnected pores. *(A) Representative confocal microscopic images of a [4% (wt/vol)] LMW HAGM cryogel of which the walls are stained with rhodamine-labeled poly-L-lysine (left) and in bright field (right). (B) Pore size of [4% (wt/vol)] LMW HAGM cryogel. 30 pores of 3 different cryogels stained with rhodamine-labeled poly-L-lysine were measured. (C) Representative scanning electron microscopy images of a 4x4x1 mm³ [3% (wt/vol)] HMW HAGM cryogel. Scale bar equals 1 mm (top) and 100 µm (bottom). (D) Pore connectivity of a [4% (wt/vol)] LMW HAGM cryogel. (E) Representative image of cryogel localization before and after injection through a 16G needle. (B,D) Values represent mean ± SEM.*

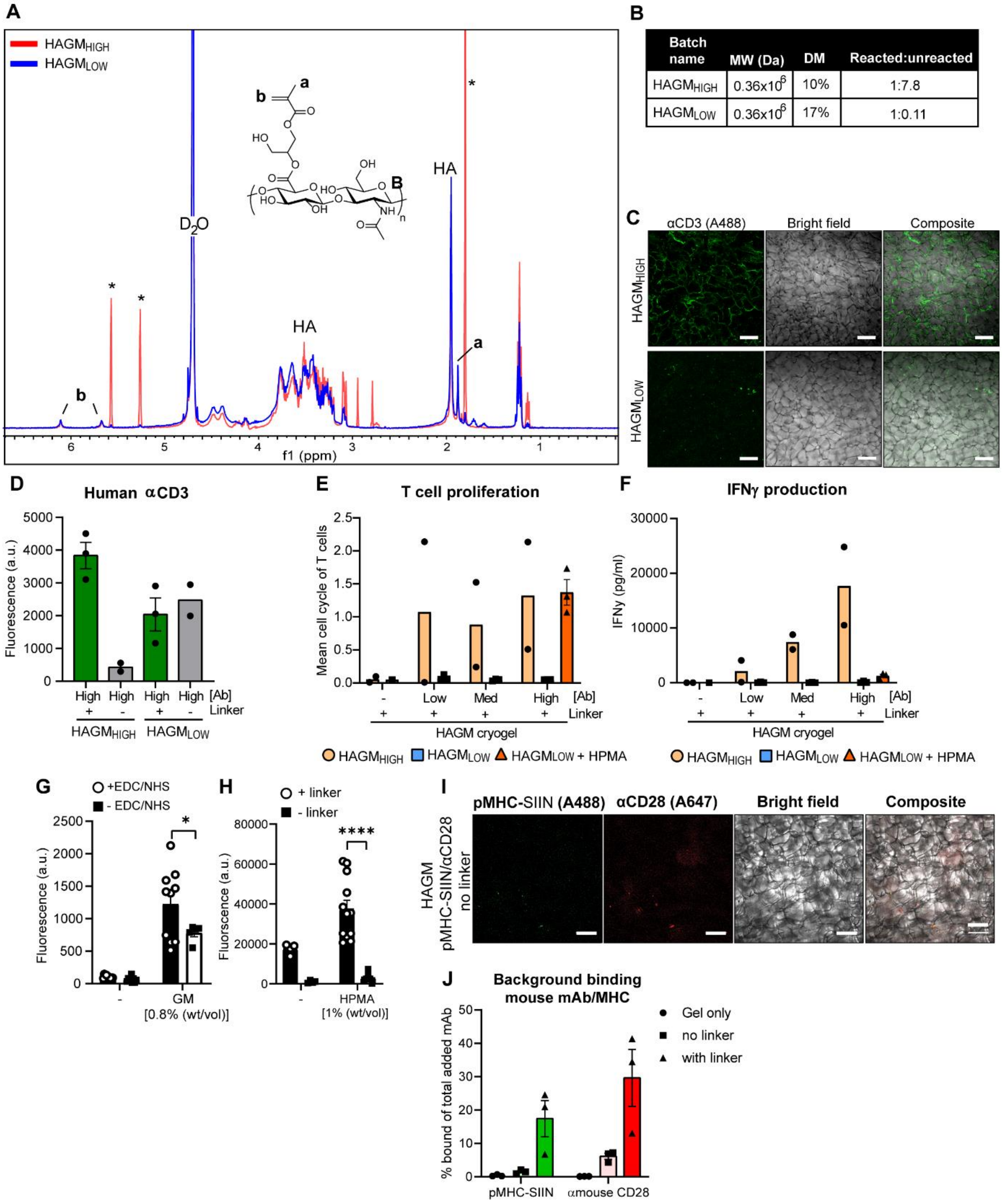

Supplemental Figure 2. Comonomers enable biomolecule labeling of HAGM cryogels. *(A) ^{1}H NMR spectra of LMW $HAGM_{LOW}$ and $HAGM_{HIGH}$ ([1% (wt/vol)] in deuterated water (D_2O)). The degree of methacrylation (DM) was determined based on the ratio of the integrals for HA protons to the protons of GM (δ5.7 and 6.1 ppm). Stars indicate the presence of unreacted GM. (B) Overview of the DM of two LMW HAGM polymer batches. MW = molecular weight. (C) Representative confocal microscopic images of [4% (wt/vol)] LMW HAGM cryogels of 2 batches labeled with high amounts of αhCD3-A488. Scale bar equals 100 µm. (D) Fluorescence quantification of LMW HAGM cryogels labeled with αhCD3-A488. (E,F) Primary human pan T cells were stimulated with LMW cryogels of $HAGM_{HIGH}$ or $HAGM_{LOW}$ or $HAGM_{LOW}$ where HPMA was added as a comonomer at [0.8% wt/vol)]. Cryogels were labeled with varying densities of αhCD3-A488 and αhCD28-A647, and the mean proliferation cycle (E) after 72 hrs and IFNγ production (F) after 24 hrs were determined. (G) Fluorescence quantification of [4% (wt/vol)] HAGM LMW cryogels labeled with amine-Cy5 linker. (H) Fluorescence quantification of [3% (wt/vol)] HAGM HMW cryogels labeled with DBCO-Cy5 linker. (I) Representative confocal microscopic images of [4% (wt/vol)] HAGM cryogels functionalized with 9.3 µg mouse pMHC-A488 (H-2K^b SIINFEKL) and 40.4 µg mouse αCD28-A647 in the absence of the azide-linker. Scale bar equals 100 µm. (J) Quantification of bound pMHC-A488 (H-2K^b SIINFEKL) and 8.1 µg αmCD28-A647 plotted as % bound of total added,*

1.8 µg pMHC-A488 (H-2K^b SIINFEKL) and 8.1 µg αmCD28-A647. Biomolecules were added either in the absence or presence of the azido-propylamine linker. (D) n=3 for + linker, n=2 for –linker in 1 independent experiment. (E,F) $HAGM_{HIGH}$ n=2 in 2 independent experiments, $HAGM_{LOW}$ n=3 in 3 independent experiments and $HAGM_{LOW}$ + HPMA n=3 in 1 independent experiment. (G,H) n=4-12 in 2-3 independent experiments. (J) n=3 in 2 independent experiments. (D) Data was analyzed for statistical significance with a Kruskal Wallis test and Dunn's correction on log-transformed data. (E,F,H) Data was analyzed using a two-way ANOVA with a Tukey's or Sidak's correction.

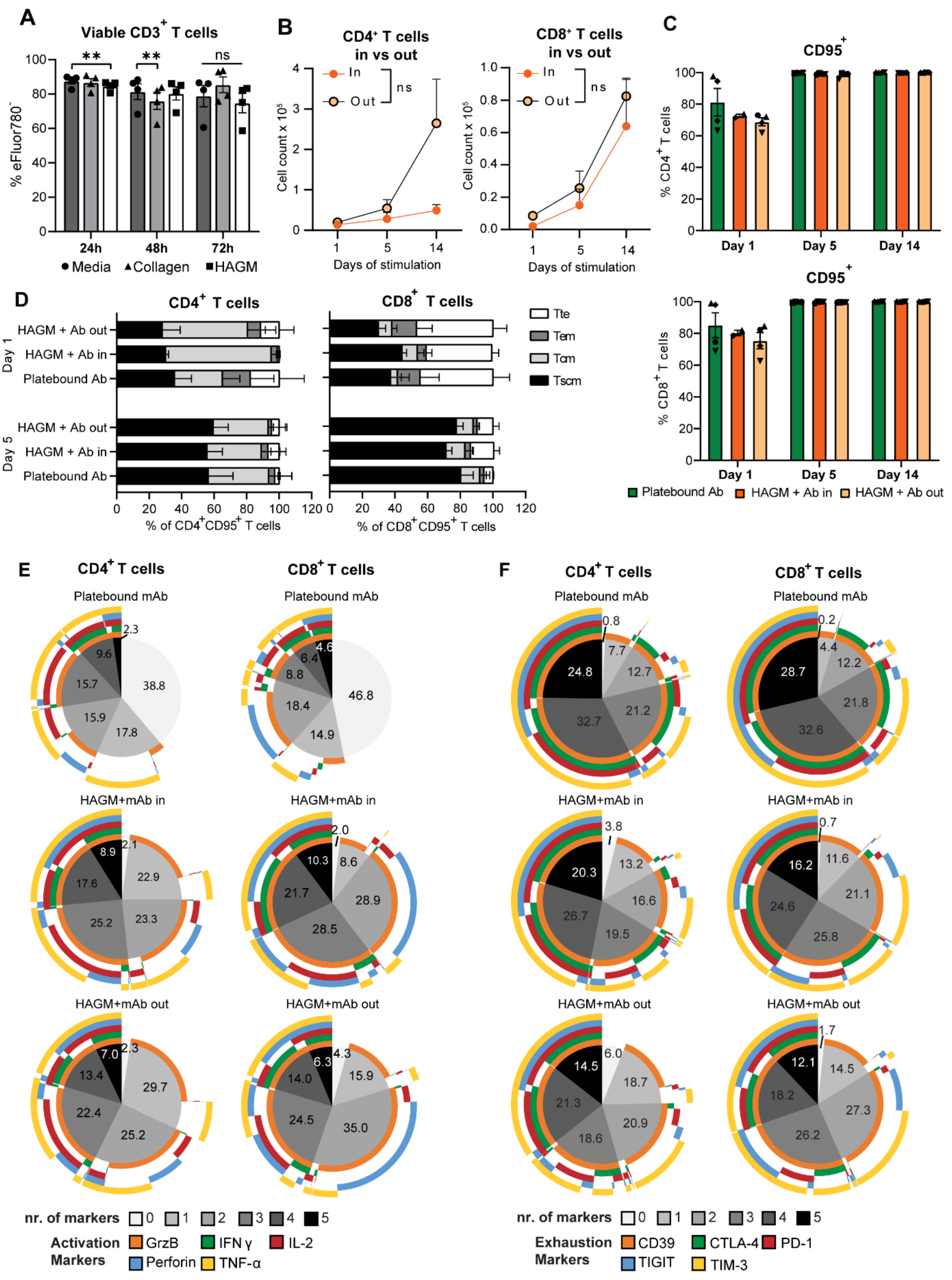

Supplemental Figure 3. HAGM cryogels support T cell viability and expansion. *(A) The percentage of eFluor780- viable total CD3+ T cells after 24, 48 or 72 hrs culturing in medium, 3D collagen gels or HAGM cryogels. (B) Human CD4+ and CD8+ T-cell expansion inside and ouside of the HAGM cryogels during 2-week culture. (C) Percentage of CD95+ CD4+ and CD8+ primary human T cells following 1, 5 and 14 days of culture. (D) Memory phenotype of CD4+ T cells and CD8+ T cells at days 1 and 5 of culture. Memory phenotype following the linear T-cell differentiation model, according to which T cells differentiate from Tscm*

(CD95$^+$CCR7$^+$CD45RA$^+$) > Tcm (CD95$^+$CCR7$^+$CD45RA$^-$) > Tem (CD95$^+$CCR7$^-$CD45RA$^-$) > Tte (CD95$^+$CCR7$^-$CD45RA$^+$). Pie charts of the (E) the effector phenotype of CD4$^+$ and CD8$^+$ T cells stimulated for 5 days or the (F) the exhaustion phenotype of CD4$^+$ and CD8$^+$ T cells stimulated for 14 days. Grey pie chart with numbers represents the percentage of cells expressing 0 to 5 effector or exhaustion markers. The colored arcs represent the 5 different effector or exhaustion markers and their percentual expression. (A) n=4 in one experiment, data analyzed with two-way anova with Tukey's correction. (B-D) n=4-6 in 2-4 independent experiments. (B) Cell counts were evaluated on day 14 with a Friedman test with a Dunn's correction. (C) Each symbols represents a healthy individual donor. (E,F) n=4 in 2 independent experiments.

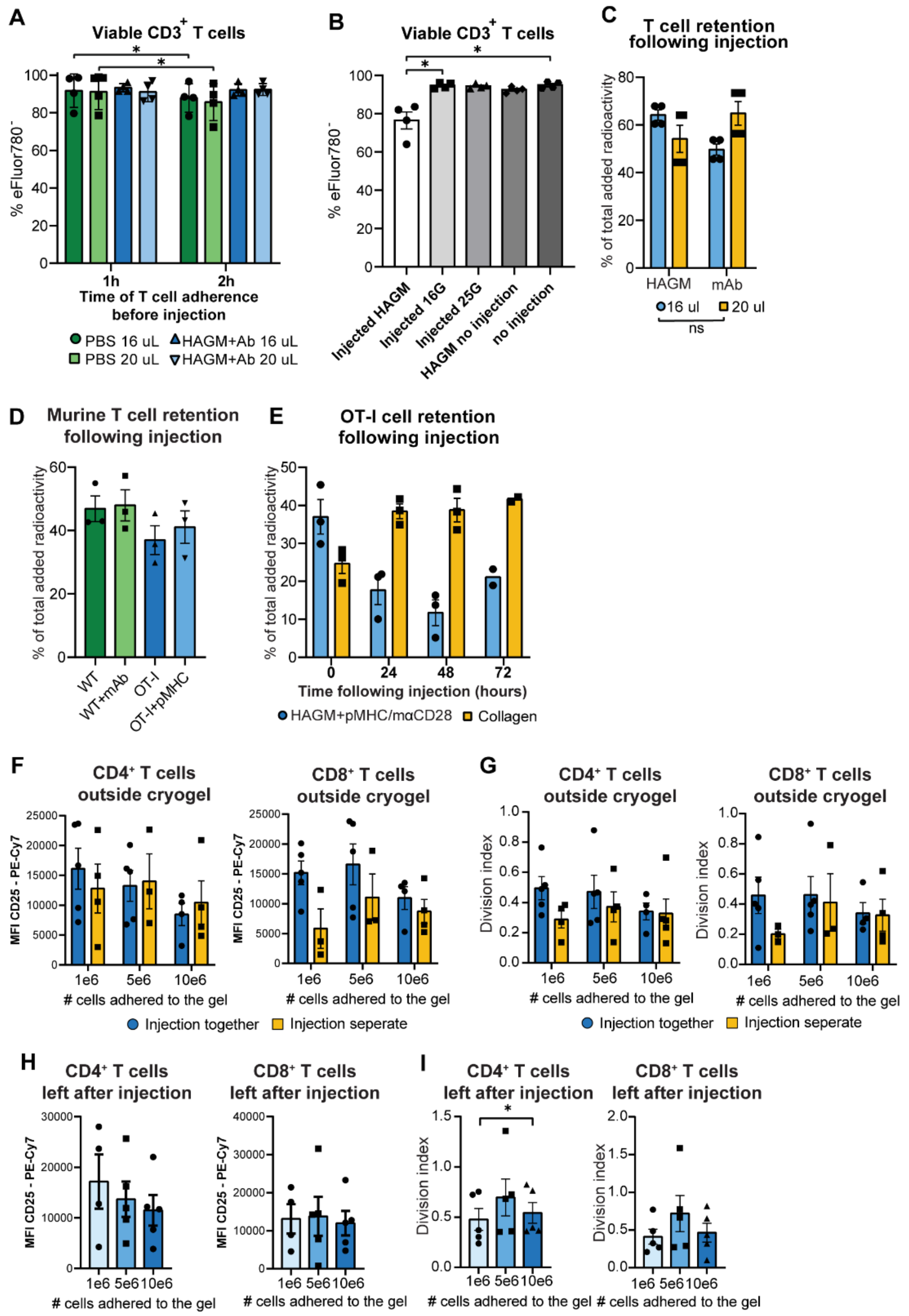

***Supplemental Figure 4. T cell retention is not influences by adherence volume of mAb presence.** (A) Viability of $0.5 \cdot 10^6$ human T cells following adherence of 1hr or 2hrs in 16 or 20 µL in PBS or in HAGM cryogels with αCD3+αCD28 Ab. (C) Human total CD3$^+$ T cells were radioactively labeled with indium oxine, adhered to the HAGM cryogels and injected into collagen matrices. The percentage retention of $0.5 \cdot 10^6$ human T cells in HAGM cryogels following injection after adherence of cells in 16 or 20 µL PBS is shown. (D) Murine WT and OT-I CD8$^+$ T-cell retention in HAGM cryogels following injection of $1 \cdot 10^6$ cells in HAGM with or without signals: murine αCD3+αCD28 for WT cells and pMHC+αCD28 for OT-I T cells. (E) Percent of $1 \cdot 10^6$ murine*

OT-I T cell retention in HAGM cryogels with pMHC-SIINFEKL+αCD28 mAb and collagen matrix following injection for 72 hrs. (F) CD25 expression and (G) division index of $CD4^+$ and $CD8^+$ human T cells retrieved from outside the HAGM scaffolds that were injected and either cultured together or separate. (H) CD25 expression and (I) division index of $CD4^+$ and $CD8^+$ T cells cultured for 3 days in a separate well from the injected HAGM scaffold. (A,C) n=4 in 2 independent experiments. (B) n=4 in 1 independent experiment. (D-E) n=2-3 in 2 independent experiments. (F-I) n=5 in 4 independent experiments. (A,C) Data evaluated with two-way ANOVA with Tukey or Bonferroni correction. (B,D, H-I) Data evaluated with one-way ANOVA with Tukey correction. (F-G) Data evaluated with mixed-effects analysis with a Tukey correction.

A

Human	FITC	PE	PerCP-Cy5.5/BB700	PE-Cy7	APC/A647	APC-Cy7	BV421	BV510
Memory	CD8			CD4	CCR7	eFluor780	CD95	CD45RA
Effector	CD4	GrzB	IL-2	TNF-α	Perforin	eFluor780	IFNγ	CD8
Exhaustion	CD8	CTLA-4	TIGIT	CD4	TIM-3	eFluor780	CD39	PD-1

B

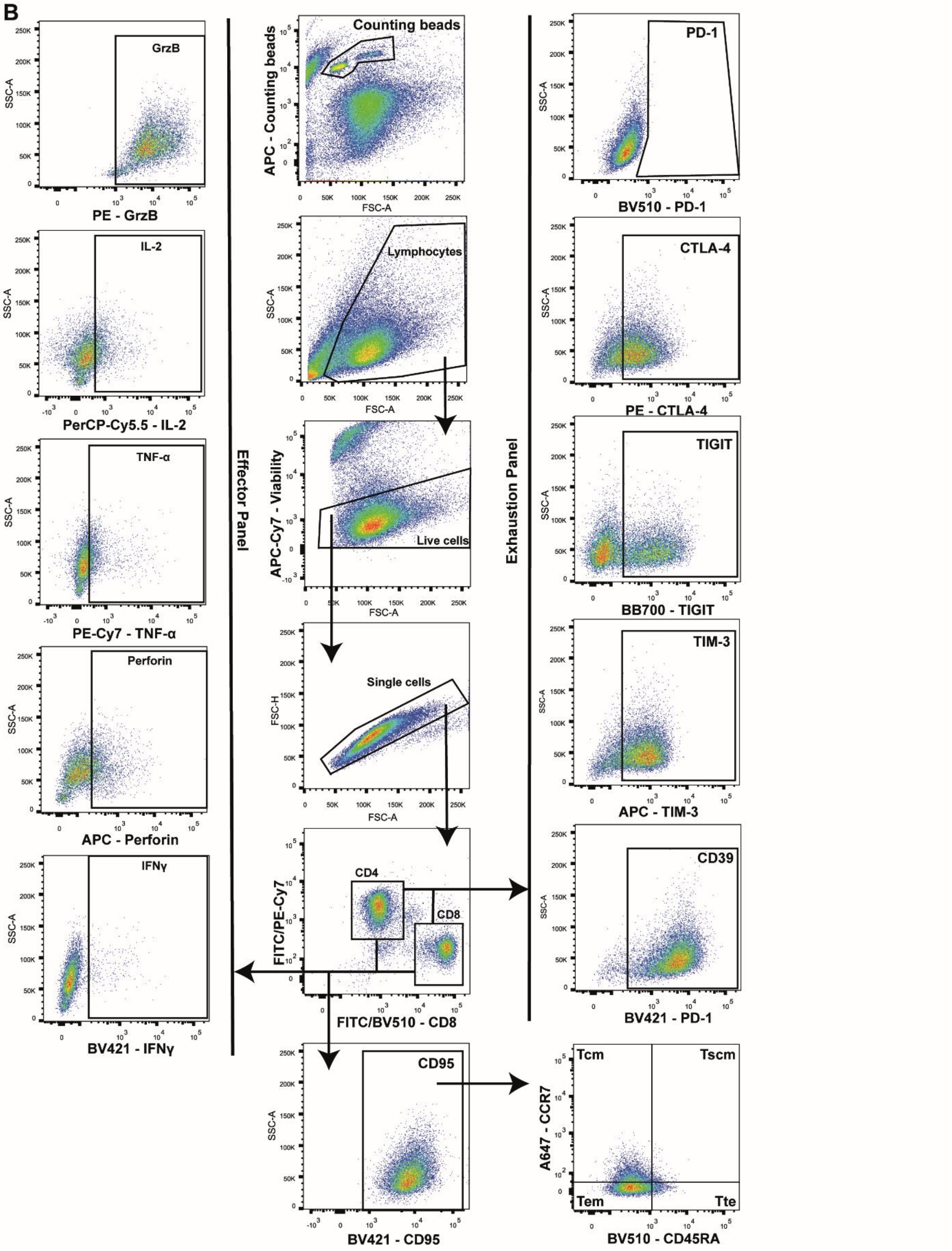

Supplemental Figure 5. Human FACS panels and gating strategy. *(A) FACS panels to determine the memory, effector, and exhaustion phenotype of human total CD3⁺ T cells. (B) Gating strategy for the memory, effector and exhaustion panels. Gating was based on fluorescence minus one (FMO) controls and negative samples.*

A

H-2K^b Heavy chain

MGPHSLRYFV TAVSRPGLGE PRYMEVGYVD DTEFVRFDSD AENPRYEPRA RWMEQEGPEY
WERETQKAKG NEQSFRVDLR TLLGYYNQSK GGSHTIQVIS GCEVGSDGRL LRGYQQYAYD
GCDYIALNED LKTWTAADMA ALITKHKWEQ AGEAERLRAY LEGTCVEWLR RYLKNGNATL
LRTDSPKAHV THHSRPEDKV TLRCWALGFY PADITLTWQL NGEELIQDME LVETRPAGDG
TFQKWASVVV PLGKEQYYTC HVYHQGLPEP LTLRWELPET GG

Human b2m

MARSVTLVFL VLVSLTGLYA IQKTGQIQVY SRHPPENGKP NILNCYVTQF HPPHIEIQML
KNGKKIPKVE MSDMSFSKDW SFYILAHTEF TPTETDTYAC RVKHASMAEP KTVYWDRDM

Peptide

SIINFEKL

Supplemental Figure 6. MHC protein sequence and amounts added to the HAGM scaffolds. *(A) Protein sequences of H-2K^b heavy chain and human β2m used for refolding of pMHC complexes.*
